# Optimization of Nazarov Cyclization of 2,4-Dimethyl-1,5-diphenylpenta-1,4-dien-3-one in Deep Eutectic Solvents by a Design of Experiments Approach

**DOI:** 10.3390/molecules25235726

**Published:** 2020-12-04

**Authors:** Stefano Nejrotti, Alberto Mannu, Marco Blangetti, Salvatore Baldino, Andrea Fin, Cristina Prandi

**Affiliations:** 1 Department of Chemistry, University of Turin, Via Pietro Giuria, 7-I-10125 Torino, Italy; stefano.nejrotti@unito.it (S.N.); salvatore.baldino@unito.it (S.B.); cristina.prandi@unito.it (C.P.); 2 Department of Drug Science and Technology, University of Turin, Via Pietro Giuria, 9-I-10125 Torino, Italy; andrea.fin@unito.it

**Keywords:** Nazarov reaction, Design of Experiments, deep eutectic solvents, surface responding analysis, Box-Behnken design

## Abstract

The unprecedented Nazarov cyclization of a model divinyl ketone using phosphonium-based Deep Eutectic Solvents as sustainable non-innocent reaction media is described. A two-level full factorial Design of Experiments was conducted for elucidating the effect of the components of the eutectic mixture and optimizing the reaction conditions in terms of temperature, time, and substrate concentration. In the presence of the Deep Eutectic Solvent (DES) triphenylmethylphosphonium bromide/ethylene glycol, it was possible to convert more than 80% of the 2,4-dimethyl-1,5-diphenylpenta-1,4-dien-3-one, with a specific conversion, into the cyclopentenone Nazarov derivative of 62% (16 h, 60 °C). For the reactions conducted in the DES triphenylmethylphosphonium bromide/acetic acid, quantitative conversions were obtained with percentages of the Nazarov product above 95% even at 25 °C. Surface Responding Analysis of the optimized data furnished a useful tool to determine the best operating conditions leading to quantitative conversion of the starting material, with complete suppression of undesired side-reactions, high yields and selectivity. After optimization, it was possible to convert more than 90% of the model substrate into the desired cyclopentenone with *cis* percentages up to 77%. Experimental validation of the implemented model confirmed the robustness and the suitability of the procedure, leading to possible further extension to this specific combination of experimental designs to other substrates or even to other synthetic processes of industrial interest.

## 1. Introduction

Since the seminal report by Nazarov in 1942 [1], the Nazarov reaction (or Nazarov cyclization) has emerged as one of the most powerful protocols for the synthesis of structurally complex molecules containing a cyclopentenone motif. Nowadays, the Nazarov cyclization constitutes a key step in the synthesis of many industrial relevant building blocks and molecules of biological and pharmaceutical interest, such as yuzurimine-type Daphniphyllum alkaloid (+)-caldaphnidine J [2], gracilamine [3], (±)-taiwaniaquinol F [4], as well as some prostaglandin analogues with remarkable anti-inflammatory effects [5]. The Nazarov reaction consists in the electrocyclization of divinyl ketones to cyclopentenones through a 4π conrotatory mechanism promoted by Bronsted (BA) or Lewis acids (LA). Many versions of such protocol have been recently developed, both in terms of substrates suitable for cyclization [6] and promoters, including transition metals catalysts [7,8,9,10,11], organocatalysts [12,13,14,15], and UV irradiation (photo-Nazarov reaction) [16].

In this context, the development of new and improved strategies for performing the Nazarov reaction under more affordable and sustainable conditions represents a primary target for synthetic chemists as well as for engineers. It is well established that specific hydrogen bond acceptors (HBAs) and donors (HBDs), combined in the correct molar ratio, are able to form eutectic systems with peculiar chemical and physical properties, commonly known as Deep Eutectic Solvents (DESs) [17,18]. Since the first report by Abbot in 2003 [19,20] DESs have emerged as an environmentally benign alternative to the common Volatiles Organic Compounds (VOCs) [21] in synthesis [22,23], extraction and separation processes [24,25] and have found wide applications as electrodeposition agents [26], as well as stabilizing agents for antibiotics [27]. Moreover, some authors have highlighted the green perspective in designing DESs based on natural products (Natural DESs or NaDESs), usually constituted by cholinium salts or urea (HBAs) and sugars or natural acids (HBDs) [28]. Several efforts have also been made on the engineering of the intrinsic properties of DESs and analogues [29], tuning physical properties such as density, viscosity, and optical behavior [30] by combining HBAs and HBDs and developing predictive models [31]. The possibility to promote the Nazarov cyclization employing natural-derived and recyclable NaDESs as non-innocent reaction media has been recently reported [32]. Choline chloride-based NaDESs containing naturally occurring carboxylic acids (HBDs) efficiently act both as solvent and catalyst for the electrocyclization of divinyl ketones under mild reaction conditions, with remarkable effects on conversions, yields and *cis*/*trans* selectivity.

In general, when DESs are employed as unconventional reaction media in organic synthesis, unpredictable outcomes are often observed, and the relationship between the intrinsic properties of the DESs and the experimental results is quite hard to rationalize. Many authors have proposed an active role of the DES in mediating or promoting an organic transformation [23,33,34]. This specific aspect is particularly important when consistent improvements of industrially important synthetic steps can be achieved in DES, and the optimization of the reaction conditions is crucial for the scale-up purpose. In general, the employment of unconventional media or subprocesses has been exploited for pursuit of the the aim to revise already well-established syntheses with a sustainable and green chemistry approach. This includes not only the use of eutectic solvents or the classical ionic liquids [35], but also ball milling processes [36,37] as well as ultrasound [38] or microwave effects [39].

In this context, the employment of a Design of Experiments (DoE) and multivariate analysis has emerged during the last years as pivotal for optimizing and improving chemical processes. When a DoE approach is used, selected responses (physical parameters, conversion, yield, selectivity, or other measurable output) are monitored within changing selected factors (numerical or categorical). Depending on the specific design, a proper number of experiments can be designed in order to perform specific multivariate analysis on the obtained data with the aim to measure the effect of the considered factors and their combination on the responses [40]. The choice of the specific DoE is related to the rationalization of the considered system. Usually, for explorative studies aimed to gain relevant information on a chemical process [41] or, for comparing different systems [42] screening experiments are employed, while, for optimizing a specific system, Surface Responding Analysis (SRA) can be conducted. At the end, a statistical model is provided which describes the system even in terms of synergic effects. These are of particular interest for synthetic chemists as are not easily detectable by the usual trial and error approach.

**Scheme 1:**
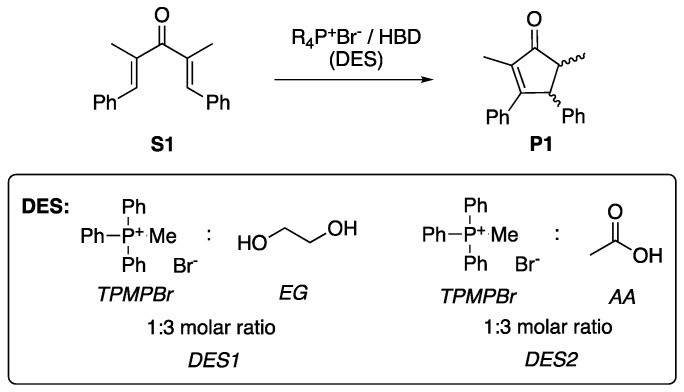
Model reaction and selected deep eutectic mixtures for the Design of Experiments (DoE) optimization of Nazarov cyclization. TPMPBr: triphenylmethyl phosphonium bromide, EG: ethylene glycol, AA: acetic acid.

As part of our ongoing research activity aimed at the development of innovative tools for the optimization and control of potentially scalable chemical reactions in sustainable media [43,44] we herein report for the first time a Design of Experiments (DoE) approach to investigate the Nazarov cyclization of a model divinyl ketone (**S1**) in two known phosphonium salt-based deep eutectic mixtures (Scheme 1) [45,46]. At first, a two-level full factorial design 2^k^ (k = factors) was used to highlight the effect of (a) temperature, time, DES type, concentration, and (b) their combination both on the conversion of the starting material and on the specific conversion of the starting material to the target cyclopentenone. Once the most suitable system was identified, a Box-Behnken design was employed followed by a Surface Responding Analysis (SRA), allowing for the development of a powerful tool with predictive ability in terms of yields and selectivity aimed at determining the best experimental conditions under different circumstances.

## 2. Results and Discussion

In order to properly estimate the reactivity of the model substrate 2,4-dimethyl-1,5-diphenylpenta-1,4-dien-3-one (**S1**) in the two selected DESs triphenylmethylphosphonium bromide/ethylene glycol 1/3 (**DES1**) and triphenylmethylphosphonium bromide/acetic acid 1/3 (**DES2**), some preliminary experiments were performed. At first, triphenylmethylphosphonium bromide (TPMPBr, Lewis acid) and acetic acid (AA, Bronsted acid) were tested independently as promoters for the Nazarov cyclization of **S1** in the presence of different solvents (Table 1).

The Nazarov cyclization of the dimethyl symmetrically substituted model substrate **S1** in the presence of Lewis or Bronsted acids affords the corresponding cyclopentenone derivatives **P1**, alongside with the methylidene cyclopentanone **P2** which is a known by-product already observed during such transformation [47].

In Table 1, the percentages of starting material and products (**P1** and **P2**) observed by ^1^H NMR after the reaction time are reported. The missing percentages in each entry are related to the presence of unidentified products in the reaction mixture. Moreover, for the product **P1**, the relative percentage of *cis* isomer is reported in the sixth column.

As shown in Table 1, the outcome of the reaction is highly dependent on the promoter as well as from the solvent employed. In general, using CPME as solvent, low percentages of starting material were converted. In the case of AA (entry 2), 22% of **P2** was recovered alongside with a mixture of uncharacterized by-products. Regarding the combination between solvent and promoter, DCE seems to be more effective using TPMPBr as catalyst (entry 2 vs. 4), while AA performs better with ethylene glycol (EG) (entry 4 vs. 6). A solubility issue was encountered in the reaction mixtures containing TPMPBr, which is slightly soluble both in CPME and in DCE. Its solubility increases in EG, resulting in a homogeneous reaction mixture, likely due to the formation of a eutectic system in the molar ratio employed (TPMPBr and EG 1/3). To the best of our knowledge, the reactions performed as indicated in entries 1, 3, and 5 in Table 1 represent the first examples of a Nazarov reaction promoted by the Lewis acid triphenylmethylphosphonium bromide. Very recently, the highly electrophilic phosphonium-based salt [(C_6_F_5_)_3_PF]^+^[B(C_6_F_5_)_4_]^−^ was employed by Vogler et al. for the Nazarov cyclization of several dienones with yields ranging from moderate to good [14].

An exhaustive rationalization of the reactivity summarized in Table 1 is challenging. AA and TPMPBr are both able to promote the Nazarov cyclization, but their activity is related to a specific combination of solvent, temperature and reaction time. Moreover, in the case of TPMPBr/EG a deep eutectic mixture is formed in situ, which is known to have different properties compared to the simple mixture of the two single components.

In this context, a deep knowledge of how to properly employ these HBAs and HBDs in terms of combinations, temperature, time and concentration of **S1** would allow us to unlock all the potential of the reactive systems (high yield and selectivity under mild conditions). Thus, the two eutectic mixtures TPMPBr/EG (**DES1**) and TPMPBr/AA (**DES2**) were considered.

A Design of Experiments (DoE) approach was envisaged in order to determine the effects of the main experimental parameters and their combinations on the Nazarov reaction outcome. In particular, the effect of temperature, reaction time, DES type, and substrate concentration were assessed by a two-level full factorial screening design of experiment 2^k^ (K = 4, 16 experiments). The Total Conversion of **S1** and the specific conversion to **P1** were considered as responses (Table 2), and 16 experiments were performed (see Supporting Information, Table S1).

The multivariate analysis of the collected experimental data allowed us to implement a model describing the influence of the considered factors on the response Total Conversion (see Supplementary Materials, Table S2). The most significant parameter to consider from the corresponding Analysis of Variance (ANOVA) is the *p*-value, obtained by testing the statistical significance of each effect by comparing the mean square versus an estimate of the experimental error. In this case, no factor or combination shows a *p* < 0.05, which would indicate a significant effect of such factor (or combination) at a confidence level of 95%. The R-Squared value of 88.756 indicates that the fitted model explains about 89% of the variability for the Total Conversion. The adjusted R-squared statistic, which is more suitable for comparing models with different numbers of independent variables, is 66.268%. The standard error of the estimate shows the standard deviation of the residuals to be 23.9071. The mean absolute error (MAE) of 11.5 is the average value of the residuals. The Durbin-Watson (DW) value, which resulted to be 1.87134 (*p* = 0.5876), means that there is no indication of serial autocorrelation in the residuals at the 5.0% significance level.

**Figure 1:**
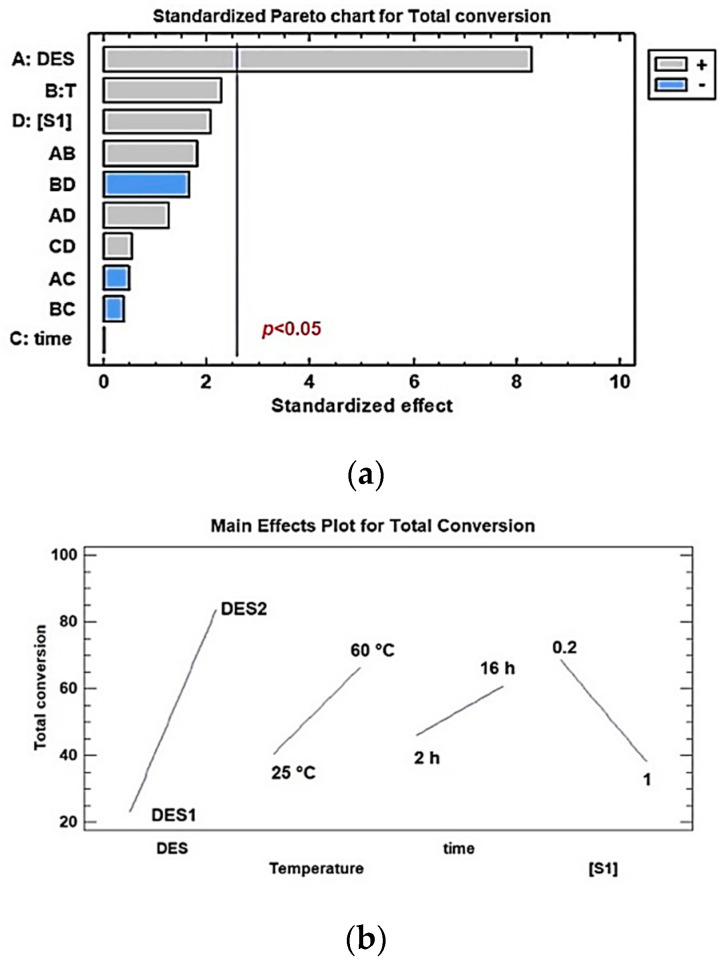
Pareto charts (**a**) and main effect plots (**b**) for the responses Total Conversion.

Additional information about the influence of the factors on the conversion of the starting product can be obtained from the Pareto chart as well as from the main effect plots (Figure 1). The Pareto chart (Figure 1a) revealed that the reactivity of 2,4-dimethyl-1,5-diphenylpenta-1,4-dien-3-one depends mostly on the type of DES. Moreover, it is interesting to notice that the temperature is more relevant than substrate concentration [**S1**] and time in determining of the Total Conversion values. Further insights can be obtained from the main effect plots (Figure 1b), where the line slope is indicative of the impact of each factor on the Total Conversion. This is provided by representing the experimental variation of the response (Y axes) as a function of the variation of each factor. It is possible to appreciate that the more impacting parameter is the DES type, followed by concentration, temperature (T), and reaction time.

The ANOVA statistic of the collected experimental data and referred to the Conversion to **P1** response gave slightly better results in terms of main statistic descriptor than the ones referred to the Total Conversion (see Supplementary Materials, Table S3). The R-Squared statistic indicates that the fitted model explains 90.2149% of the variability. The adjusted R-squared statistic is 70.6447%. The standard error of the estimate shows the standard deviation of the residuals to be 22.0695. The mean absolute error (MAE) of 10.7266 is the average value of the residuals.

**Figure 2:**
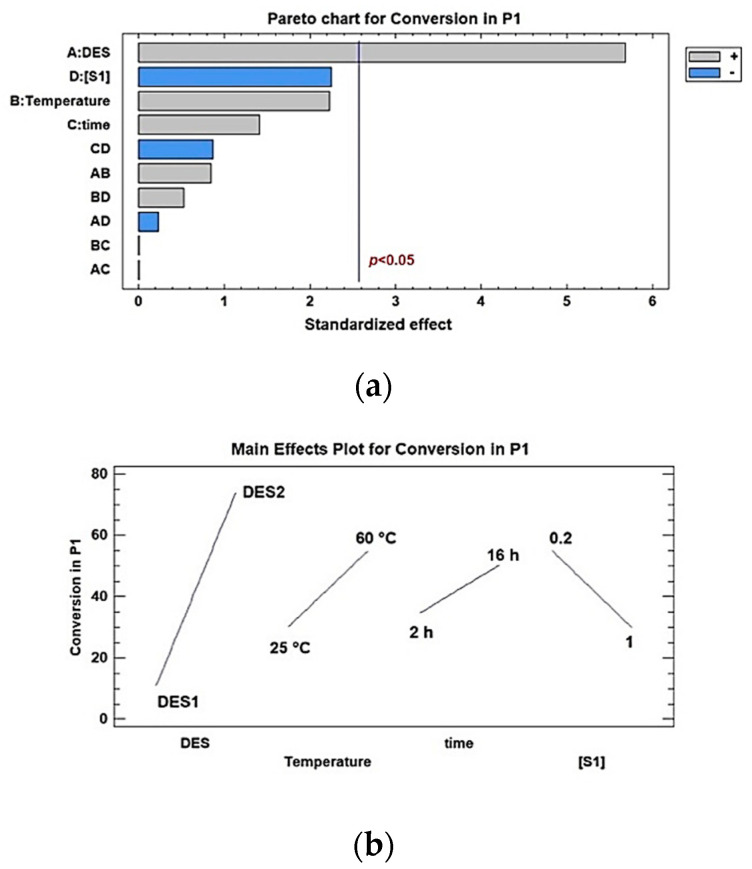
Pareto charts (**a**) and main effect plots (**b**) for the responses Conversion in **P1**.

In order to provide a detailed description of the system, the corresponding Pareto chart and the main effect plot are reported in Figure 2. The Pareto chart (Figure 2a) shows that the conversion of the starting material **S1** in the target compound **P1** is strongly influenced by the type of DES employed as solvent. This specific effect has an associated *p*-value of 0.0024 (see Supplementary Materials, Table S3) and is thus affordable at the 95% of confidence level.

The effects of the other factors and their combinations appear to have a low effect on the Total Conversion values. The main effect plot (Figure 2b) partially confirms the outcome of the Pareto chart, especially in terms of the bigger influence of the DES type. Looking at the specific impact of time, concentration, and temperature, the first one influences the conversion of **S1** in **P1** with respect to the other two factors less.

From the multivariate statistics analyses reported above, it is evident that the **DES2**, composed of TPMPBr and AA, is more performant in terms of specific conversion of **S1** in **P1**. This system was thus subjected to a further multivariate analysis in order to build a statistical tool which can be used for optimizing and engineering the system. A Box–Behnken Design (BBD) and Surface Responding Analysis (SRA) were employed for such a purpose. This specific approach has already been demonstrated to be successful for the optimization of industrial relevant processes [48] for example, for the extraction of pectin from sunflowers [49] for the industrial mercerization of cellulose [50] for the production of bio-lubricants from waste cooking oils [51] for wastewater treatment [52] and grinding processes [53].

Thus, the optimization of the main relevant conditions for the Nazarov cyclization of **S1** using **DES2** as reaction media (temperature, time, amount of DES) was envisaged. The procedure was targeted to maximize the responses Total Conversion, Conversion in **P1**, and the selectivity expressed as a percentage of *cis* isomer (Table 3). The concentration of the substrate S1 in DES is herein expressed as the amount of DES. As BBD is usually employed for optimizing industrial processes, where scale-up issues are relevant, the indication of how much DES is necessary to transform a determined amount of starting material should better describe the process.

According to the single block BBD, 15 experiments were performed, and the multivariate statistical analysis of the collected experimental data was also conducted (see Supporting Information, Tables S4 and S5).

The model implemented through the Box-Behnken design fits well for all the three responses considered (*p*-values < 0.05). The R-squared, which indicates the percentage of variance explained by the model, ranges from 91.64% to 97.91%, confirming the suitability of the model for describing the Nazarov cyclization of **S1**.

**Figure 3:**
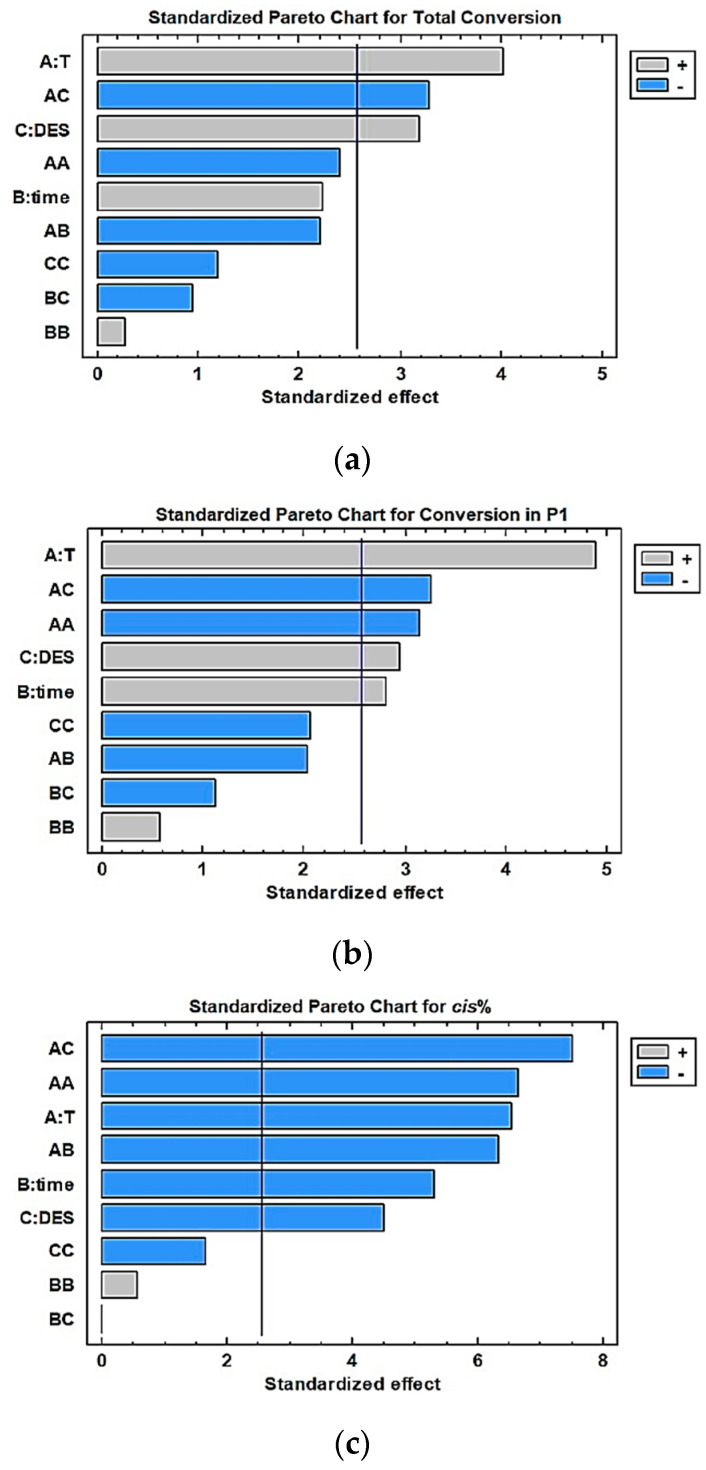
Standardized Pareto charts for Total Conversion (**a**), Conversion in **P1** (**b**) and *cis*-selectivity (**c**).

A punctual description of the behavior of the system can be achieved through the analysis of the Pareto charts for the Total Conversion, the Conversion in **P1**, and for the selectivity (Figure 3). The analysis of the relevant factors which determine the selectivity of the Nazarov cyclization of **S1** is quite complex as neither a single factor nor a combination of parameters displays a predominant effect on *cis*%. Many factors and combinations influence the *cis*% with the following order: combination between temperature and DES amount (AC) > temperature (A) > combination between temperature and time (AB) > time (B) > DES amount (C). If the influence of temperature, time and their combinations on the percentage of *cis*-2,5-dimethyl-3,4-diphenylcyclopent-2-en-1-one is in some way expected, the influence of the concentration of the starting material is something that deserves more attention. It is recurrent both in the DoE performed and for all the considered targets a strong influence of the substrate concentration in the DES. The origin of the proved relationship between concentration and the reaction outcome is hard to rationalize. It can be related to two main aspects: the homogeneity of the system, which become heterogeneous when 0.2 mmol of substrate are suspended in 0.2 g of DES, and to the molar ratio between substrate and DES. In fact, the substrate itself as well as the reaction products can act as hydrogen bond acceptors, influencing the structure and properties of the DES, and turning the original binary system in a ternary or quaternary one. As an example, in the case of water, it has been largely assumed that it exists a limit of water concentration (between 20 wt% and 30 wt% for different DESs) [54] beyond which the eutectic evolves into a classic solution. In the case herein discussed, the higher concentration of **S1** considered (1 mmol for 1 g of DES) corresponds to about the 54% in moles [55] which revealed to be detrimental for the correct proceeding of the reaction.

In order to maximally exploit the data acquired through the BBD, a Surface Responding Analysis (SRA) was conducted. The target of such multivariate statistics was to maximize the three responses (Total Conversion, conversion in **P1**, and *cis*%) at the same time. The goal of such a process is to find the best compromise between temperature, time, and DES amount for obtaining the higher yield in P1 with the maximum selectivity (*cis*%) (Table 4). In order to properly implement the statistical model, the results were expressed as function of a so-called desirability which is an equation generated from the experimental data that express the aimed maximization of the three responses described in Table 4.

The desirability function implemented ranges from 0 (Total Conversion = 22%, Conversion in **P1** = 11%, and *cis*% = 67%) to 1 (Total Conversion = 100%, Conversion in **P1** = 94%, and *cis*% = 79%). The experimental limits for the considered factors are also reported in Table 4. The statistical model has been fit to the three response variables, which show *p*-values < 0.05, indicating that the model as fit is statistically significant. In addition, the R-squared statistic range from 91.64% to 97.91%, confirming the goodness of the model (See Supplementary Materials, Table S6).

**Figure 4:**
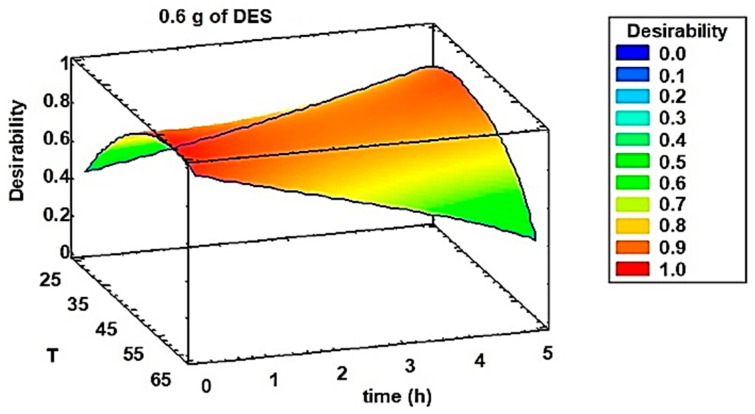
Desirability plot for 0.2 mmol of **S1** treated with 0.6 g of **DES2.**

Once the statistical robustness of the implemented model has been confirmed, the behavior of the system can be represented as a surface function of the desirability equation. As shown in Figure 4, the concentration of the substrate has been fixed at 0.2 mmol of **S1** for 0.6 g of **DES2**, as it turned out to be the more appropriate to achieve the best results in terms of desirability. On the other side, the time performances of the reaction can be evaluated by varying the temperature and the time along the X and Y axes.

The surface obtained reveals two possible routes to reach the maximum desirability: performing the Nazarov cyclization of **S1** in a short reaction time (1 h) at a temperature between 55 and 65 °C or lowering the temperature to 30–35 °C and running the reaction for longer time (at least 5 h). These two sets of reaction conditions would afford, according to the statistical model, a quantitative conversion of **S1**, with a specific conversion in **P1** between 90% and 95%, and a cis% between 75% and 80%. Experiments conducted at 60 °C during 1h (0.6 g of DES) afforded a quantitative conversion of S1, a conversion in **P1** of 91%, and a *cis%* of 77%, while experiments conducted at 25 °C gave, respectively, a quantitative conversion, 84% con **P1** conversion, and 77% of *cis%* (Table S4).

In order to avoid any undesired reactions, prolonged reaction times should be weighted by an opportune decreasing of temperature and vice-versa.

In principle, both of the experimental conditions suggested by the SRA analysis are suitable and the discerning of the most affordable is related to specific needs (time or energy saving).

The model herein presented, resulting from two consecutive DoEs, represents a powerful instrument for determining the best experimental condition for the Nazarov cyclization of **S1** in the **DES2**. Such a tool can be easily employed at the industrial level for engineering the reaction by defining the targets which are needed in the specific context.

As a last step of the optimization procedure, an experimental validation of the SRA was conducted. The prediction ability of the model toward the Total Conversion and the Conversion in **P1** was verified by performing the Nazarov cyclization at 50 °C in 1 h with an amount of DES of 0.6 g. Then, the experimental results were compared with the ones calculated from the regression equations developed from the SRA for each of the responses (Equations (1) and (2) and Table 5).

Total Conversion = 51.55 + 0.9 × Temperature(1)

Conversion in **P1** = 36.7143 + 0.971429 × Temperature (2)

As shown in Table 5, the experimental validation confirms the goodness of the model which was already evaluated through the analysis of the variance. For the validation experiment, 0.2 mmol of starting material S1 were treated with 0.6 g of DES for 1 h at 50 °C.

## 3. Materials and Methods

### 3.1. General

Unless specified, all reagents were used as received without further purifications. ^1^H NMR spectra were recorded on a Jeol ECZR600 (Tokyo, Japan), in CDCl_3_, using residual solvent peak as an internal standard (CHCl_3_, ^1^H: 7.26 ppm). Multiplicity is reported as follows: s (singlet), d (doublet), t (triplet), q (quartet), quin (quintet), sext (sextet), m (multiplet), br (broad). GC-MS spectra (Agilent, Carpinteria, CA, USA) were recorded at an ionizing voltage of 70 eV. The model divinyl ketone 2,4-dimethyl-1,5-diphenylpenta-1,4-dien-3-one (**S1**) was synthesized according to a previously reported procedure [56]. Conversions and yields were determined on the crude reaction mixture using nitromethane as internal standard.

### 3.2. Preparation of DES1

Triphenylmethylphosphonium bromide (1.50 g, 4.2 mmol) and ethylene glycol (0.78 g, 12.6 mmol) were mixed in a 5 mL screw-cap vial and stirred at room temperature for 2 h. The resulting homogeneous mixture was used without further purifications.

### 3.3. Preparation of DES2

Triphenylmethylphosphonium bromide (1.50 g, 4.2 mmol) and acetic acid (0.40 g, 6.7 mmol) were mixed in a 5 mL screw-cap vial and stirred at room temperature for 2 h. The resulting homogeneous solution was used without further purifications.

### 3.4. General Procedure for the Nazarov Cyclization Reaction.

2,4-Dimethyl-1,5-diphenylpenta-1,4-dien-3-one (**S1**) (0.2 mmol) and the DES were mixed in an open screw-cap vial and stirred at the determined temperatures and times. Water (or a 1.0 M NaOH solution for **DES2**) was added and the organic layers were extracted with dichloromethane (DCM, 3 × 5 mL). The combined organic layers were dried over anhydrous Na_2_SO_4_, filtered and the solvent was evaporated under reduced pressure. The crude 2,5-dimethyl-3,4-diphenylcyclopent-2-en-1-one (**P1**) was analyzed by ^1^H NMR using nitromethane as internal standard. All the spectroscopic data were in accordance with those reported in the literature [56].

### 3.5. Statistical analysis

Full factorial and Box-Behnken designs, as well as the statistical multivariate analysis, were performed using Statgraphics Centurion 18 software (Virginia, WV, USA). The experiments were conducted in a single block and in randomized order.

## 4. Conclusions

The Nazarov cyclization of the model substrate 2,4-dimethyl-1,5-diphenylpenta-1,4-dien-3-one **S1** was optimized in deep eutectic mixtures containing triphenylmethylphosphonium bromide (TPMPBr) as hydrogen bond acceptor (HBA) and acetic acid (AA) or ethylene glycol (EG) as hydrogen bond donors (HBDs). Full and very good conversions were observed, respectively, in the presence of TPMPBr/AA, and TPMPBr/EG (82%). An unexpected synergic effect of the former HBA and HBD was observed, whereas the cyclization reaction led to different outcomes in the presence of only TPMPBr, AA, or EG. This combined effect of HBA and HBD clearly reflects the deep eutectic nature of the medium, confirming the improved properties of such systems when employed as solvents. The combination between a two-level full factorial and a Box-Behnken design allowed us to determine the best experimental conditions to achieve the best conversion of S1 into the corresponding cyclopentenone product P1 arising from the Nazarov cyclization reaction. Surface Responding Analysis of the optimized data furnished a useful tool for determining the best operating conditions in terms of the concentration of substrate, temperature and reaction time. At the end of the optimization procedure, it was possible to convert more than 90% of **S1** into **P1** with a *cis*% of 77% under mild conditions (43 °C). The Experimental validation of the model implemented confirmed the robustness and the suitability of the procedure. The multivariate statistical tools herein presented well describe the Nazarov cyclization with the system considered, and the specific model implemented is valid for the substrate S1 and for the DES2. Nevertheless, the combination between a two-level full factorial design and the Surface Responding Analysis, implemented with the factors and the responses herein considered, represents a route for optimizing any DES-promoted Nazarov cyclization.

## Figures and Tables

**Table 1:**
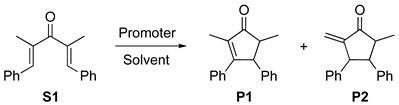
Explorative Nazarov cyclization of **S1** in the presence of TPMPBr or AA ^1^.

Entry	Promoter	Solvent	S1 (%) ^2^	P1 (%) ^2^	*cis*-P1 (%) ^2^	P2 (%) ^2^
1	TPMPBr	CPME	91	-	-	-
2	AA	CPME	41	10	60	22
3	TPMPBr	DCE	2	78	9	-
4	AA	DCE	5	17	59	30
5	TPMPBr	EG	82	62	94	-
6	AA	EG	0	30	57	22

^1^ Reaction conditions: 0.2 mmol. of **S1** in 670 mg of TPMPBr and 5.660 mmol of solvent or 340 mg of AA and 1.88 mmol of solvent, 16 h, 60 °C. CPME: cyclopentyl methyl ether, DCE: 1,2-dichloroethane, EG = ethylene glycol. ^2^ Calculated by ^1^H NMR using nitromethane as internal standard.

**Table 2:** Factors and responses considered for the full factorial screening design.

Factor	Level − 1	Level + 1	Unit
Temperature	25	60	°C
Time	2	16	h
Substrate concentration	1.0	0.2	mmol **S1**/g DES
DES	**DES1** ^1^	**DES2** ^2^	
Responses			
Total conversion	% of consumed **S1**		
Conversion to **P1**	% of **P1** formed		

^1^ TPMPBr and ethylene glycol in 1/3 molar ratio. ^2^ TPMPBr and acetic acid in 1/3 molar ratio.

**Table 3:** Factors and responses object of Box-Behnken DoE.

Factor	Level − 1 ^1^	Level + 1 ^1^	Unit
Temperature	25	60	°C
Time	1	5	h
Amount of DES	0.2	1.0	g DES for 0.2 mmol **S1**
Responses			
Total conversion	% of consumed **S1**		
Conversion to **P1**	% of **P1** formed		
Selectivity	% of *cis* isomer		

^1^ The levels + 1 and − 1 represent the range considered for each parameter. Additional points internal to these limits has been measured, see Table S4.

**Table 4:** Response variables to be measured and experimental variable factors.

Response Variables					
Name	Unit	Analyse	Goal	Impact	Sensitivity
Conv. to **P1**	%	Mean	Maximize	3.0	Medium
*Cis* selectivity	%	Mean	Maximize	3.0	Medium
Total Conv.	%	Mean	Maximize	3.0	Medium
Experimental variable factors					
Name	Unit	Type	Role	Low	High
A: temperature	°C	Continuous	Controllable	25.0	60.0
B: time	h	Continuous	Controllable	1.0	5.0
C: DES amount	g	Continuous	Controllable	0.2	1.0

**Table 5:** Explorative Nazarov cyclization of **S1** in the presence of TPMPBr or AA.

	Theoretical	Experimental
Total conversion	96.55%	96%
Conversion to **P1**	85.28%	82%

## Supplementary Materials

The following are available online.

## Abbreviations

AA	Acetic Acid
ANOVA	ANalysis Of Variance
BA	Bronsted Acid
BBD	Box–Behnken Design
CPME	CycloPentyl Methyl Ether
DCE	1,2-DiChloroEthane
DES	Deep Eutectic Solvent
DoE	Design of Experiment
DW	Durbin-Watson
EG	Ethylene Glycol
HBA	Hydrogen Bond Acceptor
HBD	Hydrogen Bond Donor
LA	Lewis Acid
MAE	Mean Absolute Error
NaDES	Natural Deep Eutectic Solvent
SRA	Surface Responding Analysis
TPMPBr	TriPhenylMethyl Phosphonium Bromide
VOC	Volatile Organic Compound
