# Supplementary material for: Optimization of Nazarov Cyclization of 2,4-Dimethyl-1,5-diphenylpenta-1,4-dien-3-one in Deep Eutectic Solvents by a Design of Experiments Approach
Source: Molecules. 2020 Dec 4;25(23):5726. doi: 10.3390/molecules25235726 (PMC7730498; doi:10.3390/molecules25235726)
Supplement: Supplementary file 1 [file molecules-25-05726-s001.pdf]

Supporting Information for

Optimization of Nazarov Cyclization of 2,4-Dimethyl-1,5-Diphenylpenta-1,4-Dien-3-One in Deep Eutectic Solvents by a Design of Experiments Approach

Stefano Nejrotti,[a] Alberto Mannu,[a] Marco Blangetti,[a]* Salvatore Baldino,[a] Andrea Fin[b] and Cristina Prandi[a]*

[a]Department of Chemistry, University of Turin, Via Pietro Giuria 7, I-10125 Turin, Italy.

[b]Department of Drug Science and Technology, University of Turin, Via Pietro Giuria 9, I-10125 Turin, Italy

Corresponding authors: alberto.mannu@unito.it ; marco.blangetti@unito.it

Table of contents

Procedure for the determination of reaction conversion and yield	S1
List of experiments and statistical analyses	S2
NMR spectra of the experiments	S6
Surface Responding Analysis	S44
References	S46

Procedure for the determination of reaction conversion and yield

Conversion and yield were determined on the crude reaction mixture by ^1H NMR on a Jeol ECZR600 using CH_3NO_2 as internal standard. The reaction crude was dissolved in $CDCl_3$, 0.5 equivalents of CH_3NO_2 (0.1 mmol, 5.4 µL) were added and the ^1H NMR of the mixture was recorded. The integral of the CH_3NO_2 peak was set to 1.5.

For the conversion, the integral of the peak corresponding to the two methyl groups in the starting material **S1** (6H, δ = 2.21 ppm)[1] was used and the following equation was applied:

$$conversion\ (\%) = \left(1 - \frac{x(\mathbf{S1})}{6}\right) \cdot 100$$

For the conversion to **P1** and **P2**, the integral of the peak corresponding to CH group α to the carbonyl group in product **P1** (1H, δ = 4.62 ppm for *cis*-P1, δ = 3.98 ppm for *trans*-P1)[1] or the integral of the peak corresponding to the terminal alkene protons in product **P2** (1H, δ = 6.26 and 5.05 ppm)[2] were used and the following equations were applied:

$$yield\ (\%) = x(\mathbf{P1}, cis + trans) \cdot 100$$

$$yield\ (\%) = x(\mathbf{P2}) \cdot 100$$

x = value of the integral

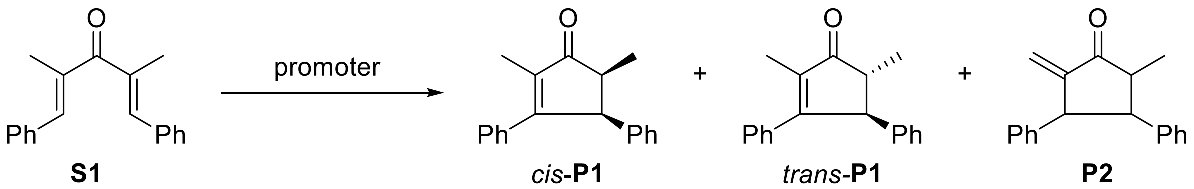

Figure S1. Main products of the Nazarov cyclization.

Table S1. List of experiments for the full factorial screening design.

Experiment	Factors				Responses	
	DES	T (°C)	Time (h)	[**S1**][a]	Total Conversion (%)[b]	Conversion in **P1** (%)[b]
1	**DES2**	60	2	0.2	100	84
2	**DES2**	25	2	1.0	24	17
3	**DES1**	60	16	0.2	82	62
4	**DES2**	60	16	0.2	100	98
5	**DES2**	60	2	1.0	100	91
6	**DES1**	60	2	0.2	29	13
7	**DES1**	60	16	1.0	13	0
8	**DES2**	60	16	1.0	100	90
9	**DES2**	25	2	0.2	94	72
10	**DES1**	25	16	1.0	9	0
11	**DES1**	25	2	0.2	13	0
12	**DES2**	25	16	1.0	51	42
13	**DES1**	60	2	1.0	6	0
14	**DES1**	25	16	0.2	31	14
15	**DES1**	25	2	1.0	2	0
16	**DES2**	25	16	0.2	100	96

[a] (mmol of **S1**)/(g of DES). [b] Determined by ^{1}H NMR using nitromethane as internal standard.

Table S2. Analysis of Variance for the response Total Conversion.

Source	Sum of Squares	Df	F-Ratio	P-Value
A: DES	14641.0	1	25.62	0.0039
B: Temperature	2652.25	1	4.64	0.0838
C: time	870.25	1	1.52	0.2721
D: [S1]	3721.0	1	6.51	0.0512
AB	196.0	1	0.34	0.5836
AC	169.0	1	0.30	0.6100
AD	2.25	1	0.00	0.9524
BC	0.25	1	0.00	0.9841
BD	225.0	1	0.39	0.5579
CD	81.0	1	0.14	0.7220
Total error	2857.75	5		
Total (corr.)	25415.8	15		

Table S3. Analysis of Variance for the response Conversion in **P1**.

Source	Sum of Squares	Df	F-Ratio	P-Value
A: DES	15687.6	1	32.21	0.0024
B: Temperature	2425.56	1	4.98	0.0760
C: time	976.563	1	2.01	0.2159
D: [S1]	2475.06	1	5.08	0.0739
AB	351.563	1	0.72	0.4343
AC	0.0625	1	0.00	0.9914
AD	27.5625	1	0.06	0.8214
BC	0.0625	1	0.00	0.9914
BD	138.063	1	0.28	0.6172
CD	370.563	1	0.76	0.4230
Total error	2435.31	5		
Total (corr.)	24887.9	15		

Table S4. List of the experiments.

Experiment	Factors			Responses		
	Temperature (°C)	Time (h)	Amount of DES[a]	Total Conversion (%)[b]	Conversion in **P1** (%)[b]	*cis* (%)[b]
1	43	1	0.2	79	66	79
2	25	5	0.6	100	84	77
3	60	3	1	100	85	67
4	60	1	0.6	100	91	77
5	60	5	0.6	100	93	67
6	43	3	0.6	100	91	77
7	25	3	1	98	78	77
8	43	3	0.6	100	91	77
9	25	1	0.6	51	42	76
10	43	1	1	100	83	76
11	43	5	0.2	100	94	77
12	25	3	0.2	22	11	73
13	43	5	1	100	89	74
14	60	3	0.2	97	82	76
15	43	3	0.6	100	90	77

[a] g of DES for 0.2 mmol of **S1**. [b] Determined by ^{1}H NMR using nitromethane as internal standard.

Table S5. Analysis of variance for the group of experiments reported in Table S4.

Model	yield	cis selectivity	conversion
Transformation	none	none	none
Model d.f.	9	9	9
P-value	0.0177	0.0011	0.0304
Error d.f.	5	5	5
Stnd. error	9.83531	0.866025	11.1041
R-squared	93.38	97.91	91.64
Adj. R-squared	81.47	94.16	76.60

Table S6. Statistical data for the implemented model.

Model	yield	*cis* selectivity	conversion
Transformation	none	none	none
Model d.f.	9	9	9
P-value	0.0177	0.0011	0.0304
Error d.f.	5	5	5
Stnd. error	9.83531	0.866025	11.1041
R-squared	93.38	97.91	91.64
Adj. R-squared	81.47	94.16	76.60

NMR spectra of the experiments

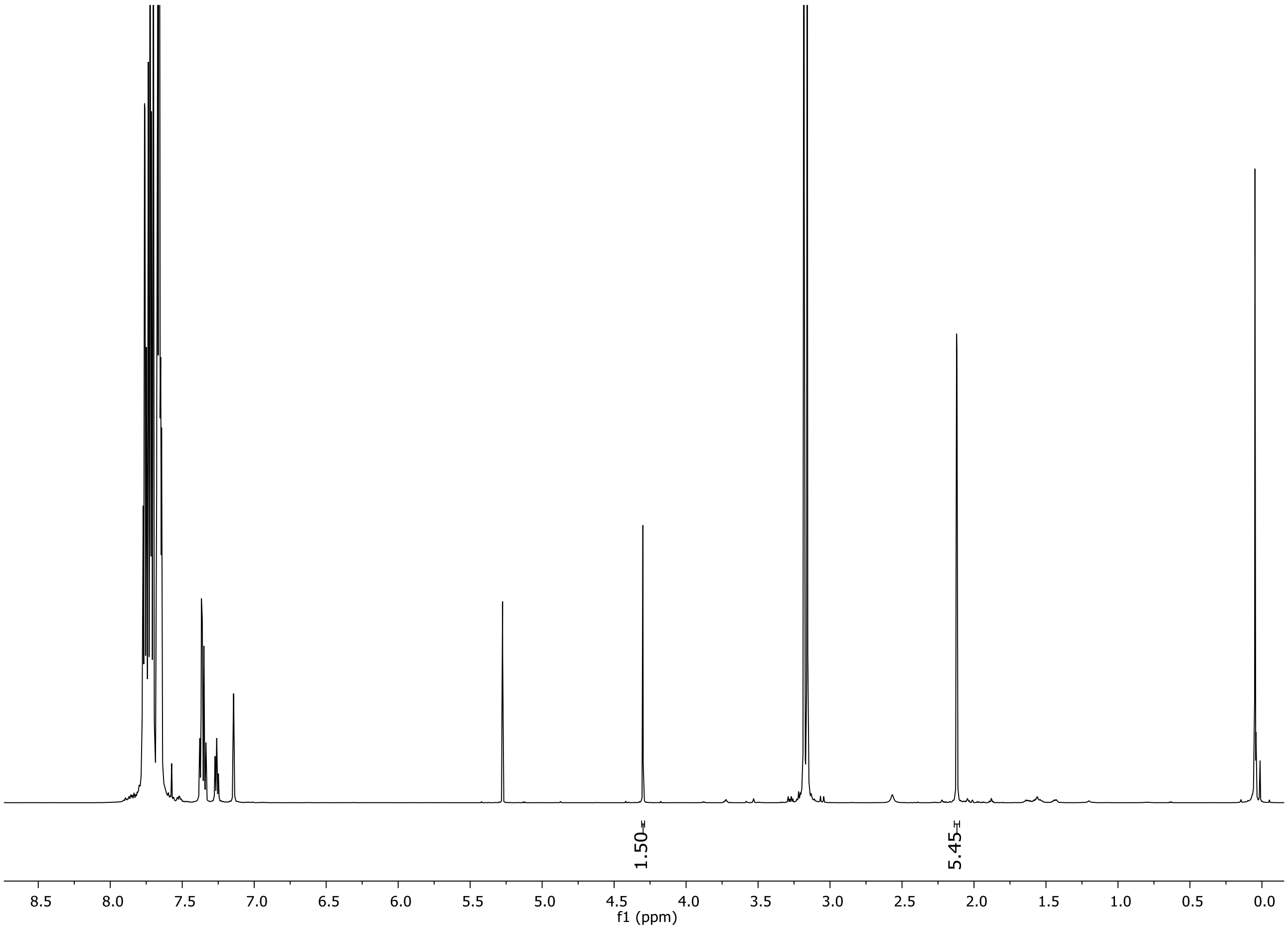

Figure S2. ^{1}H NMR of the experiment 1, Table 1.

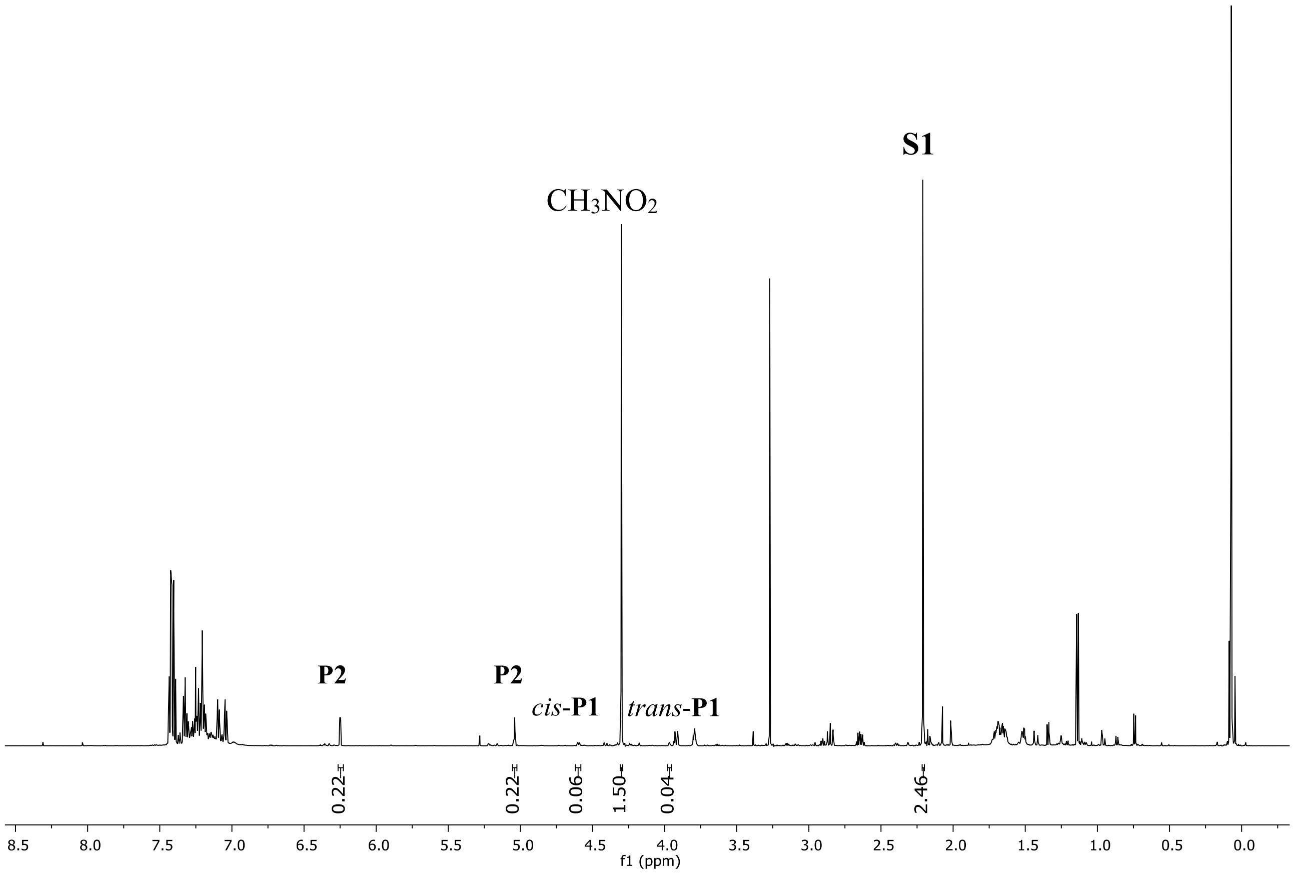

Figure S3. ^{1}H NMR of the experiment 2, Table 1.

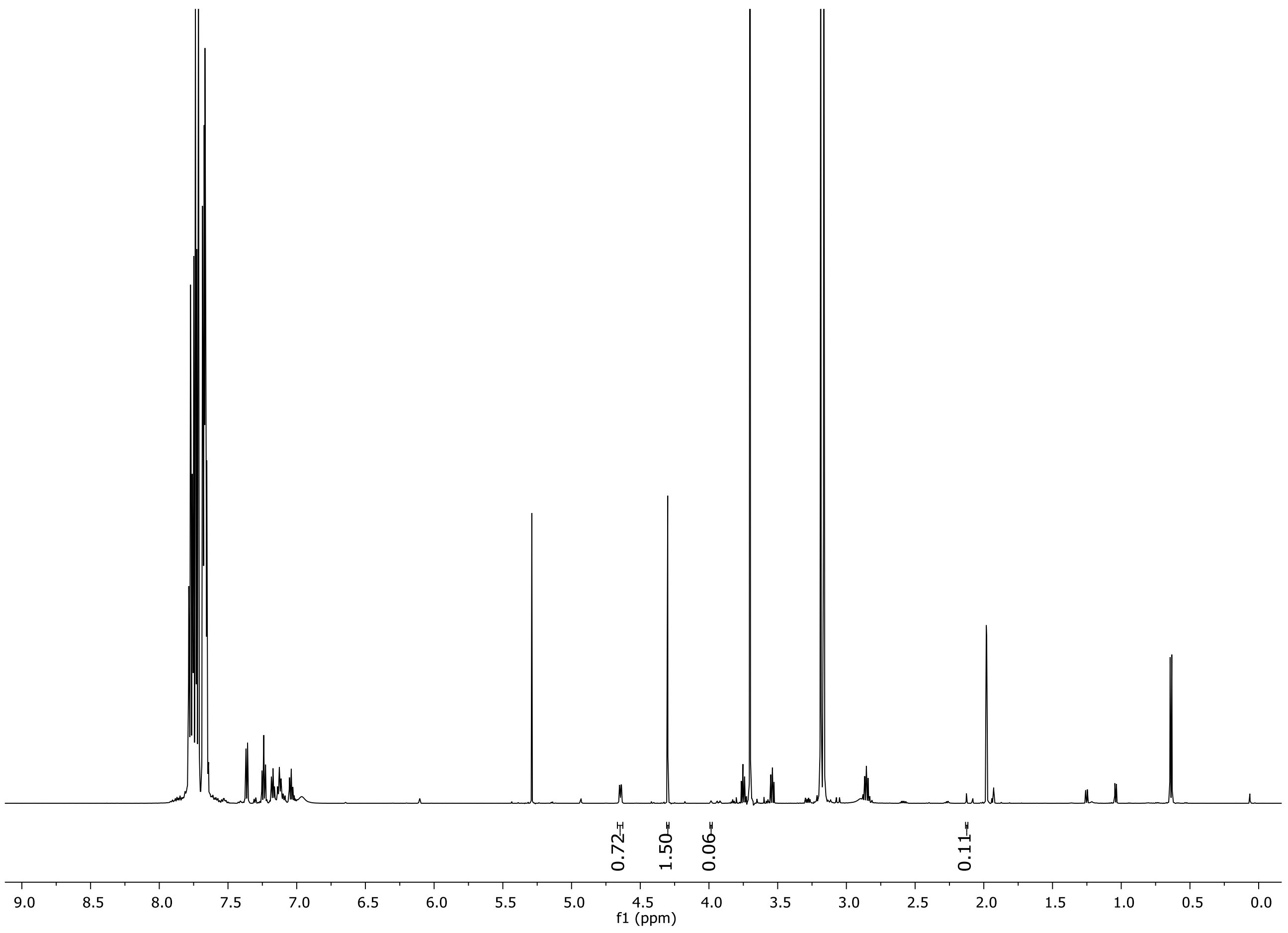

Figure S4. ^{1}H NMR of the experiment 3, Table 1.

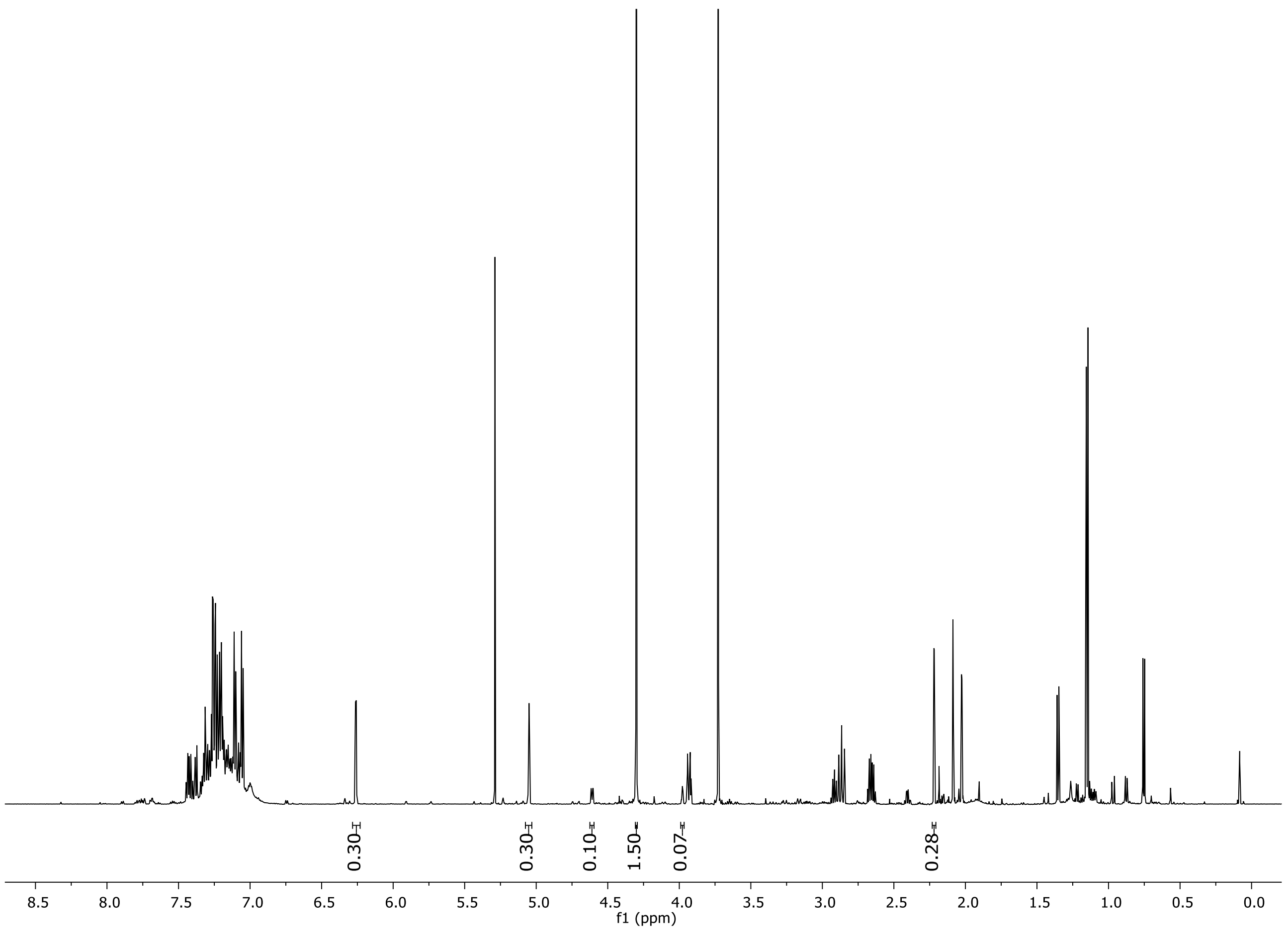

Figure S5. ^{1}H NMR of the experiment 4, Table 1.

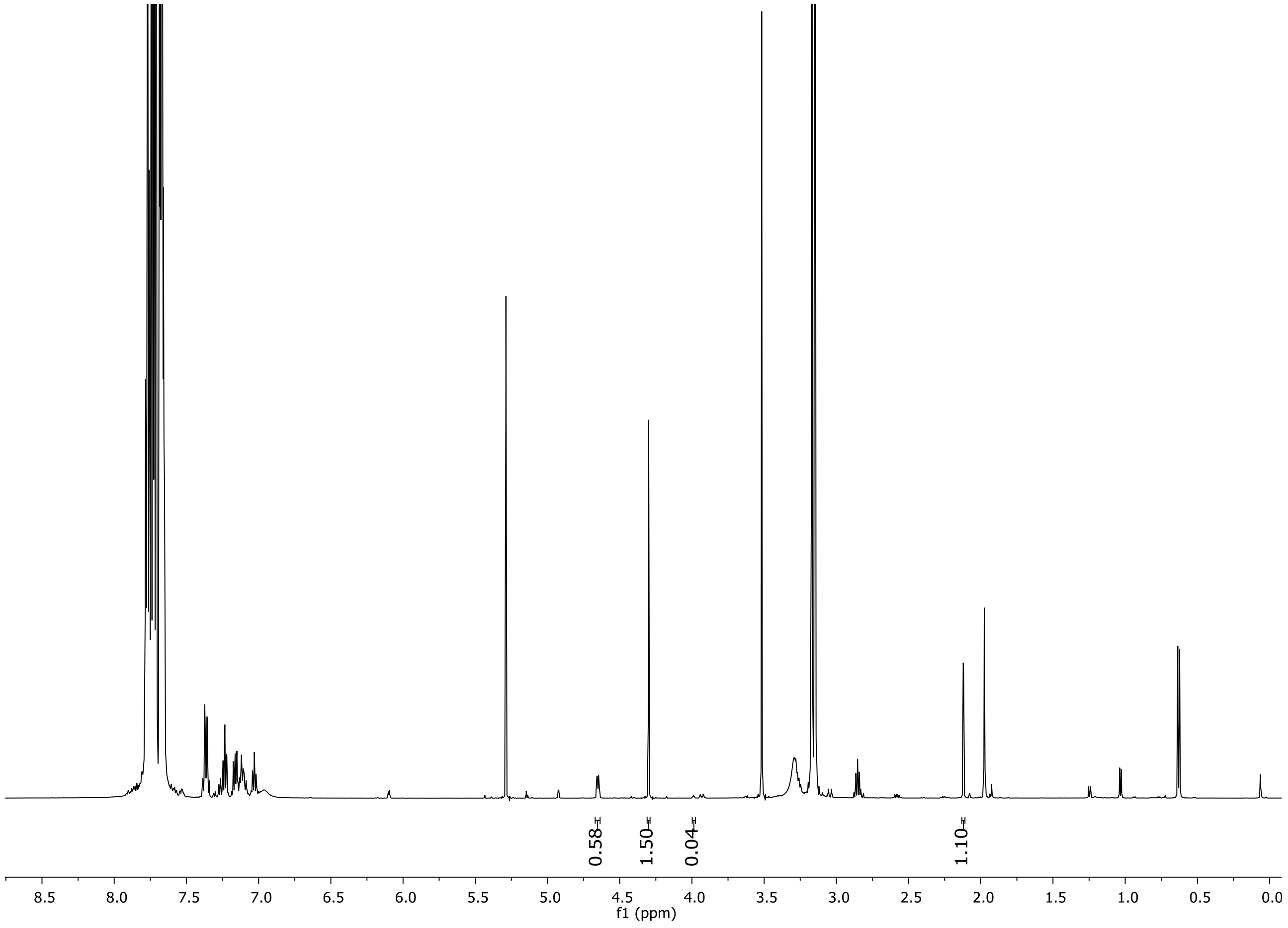

Figure S6. ^{1}H NMR of the experiment 5, Table 1.

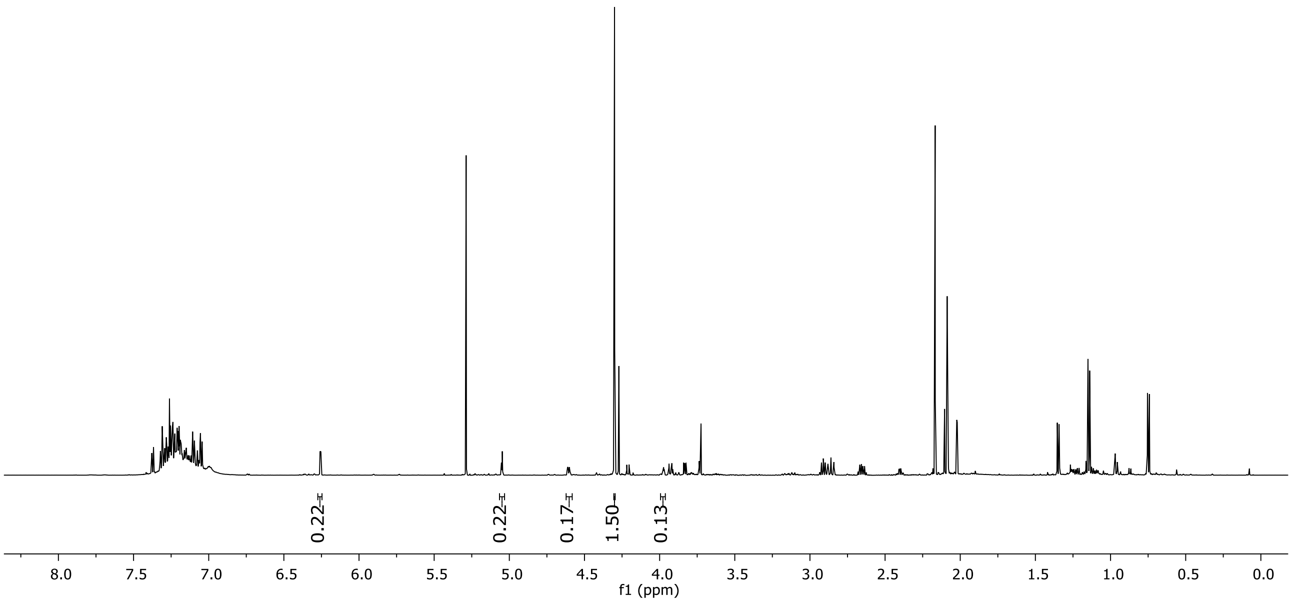

Figure S7. ^{1}H NMR of the experiment 6, Table 1.

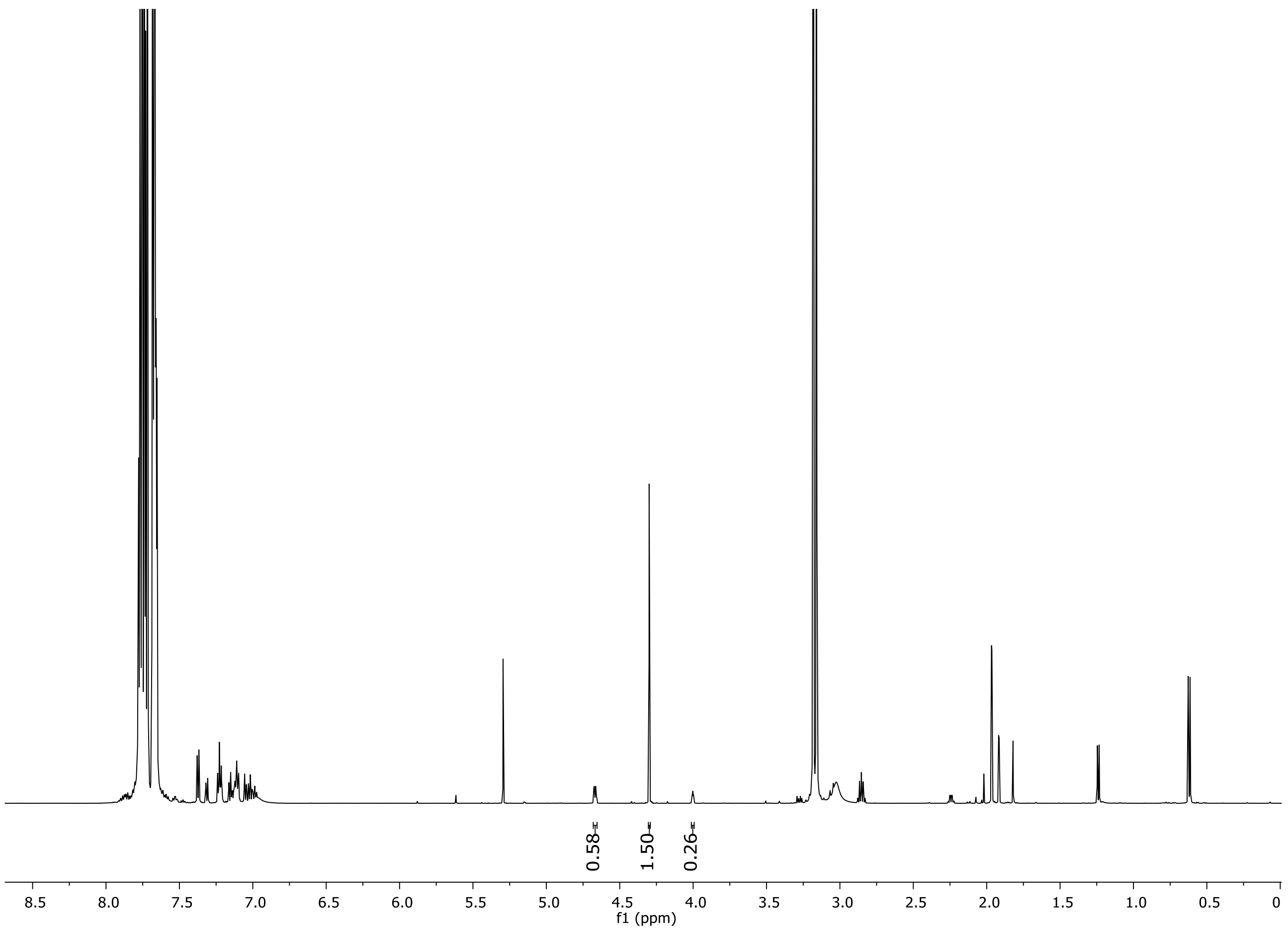

Figure S8. ^{1}H NMR of the experiment 1, Table 3.

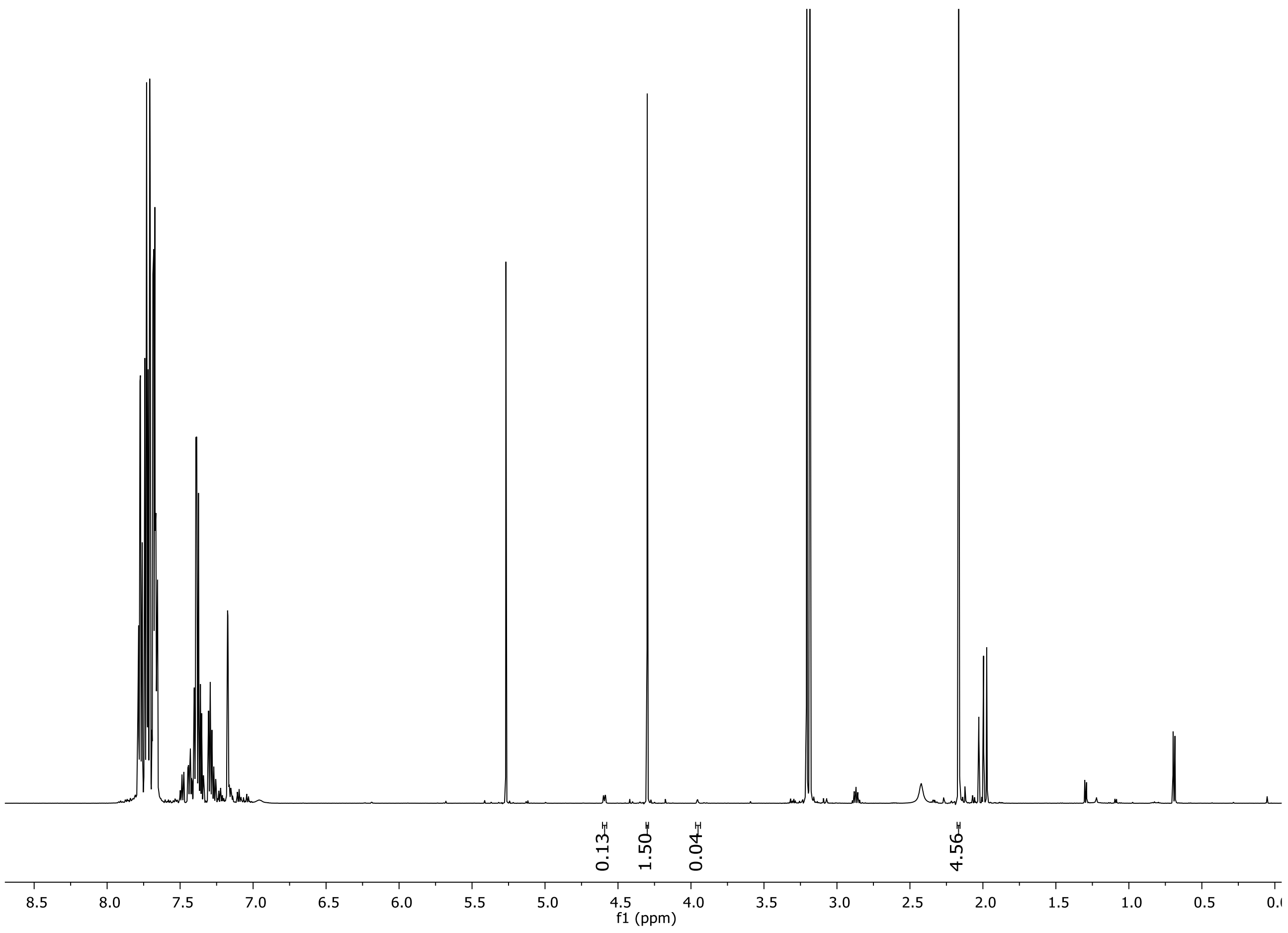

Figure S9. ^{1}H NMR of the experiment 2, Table 3.

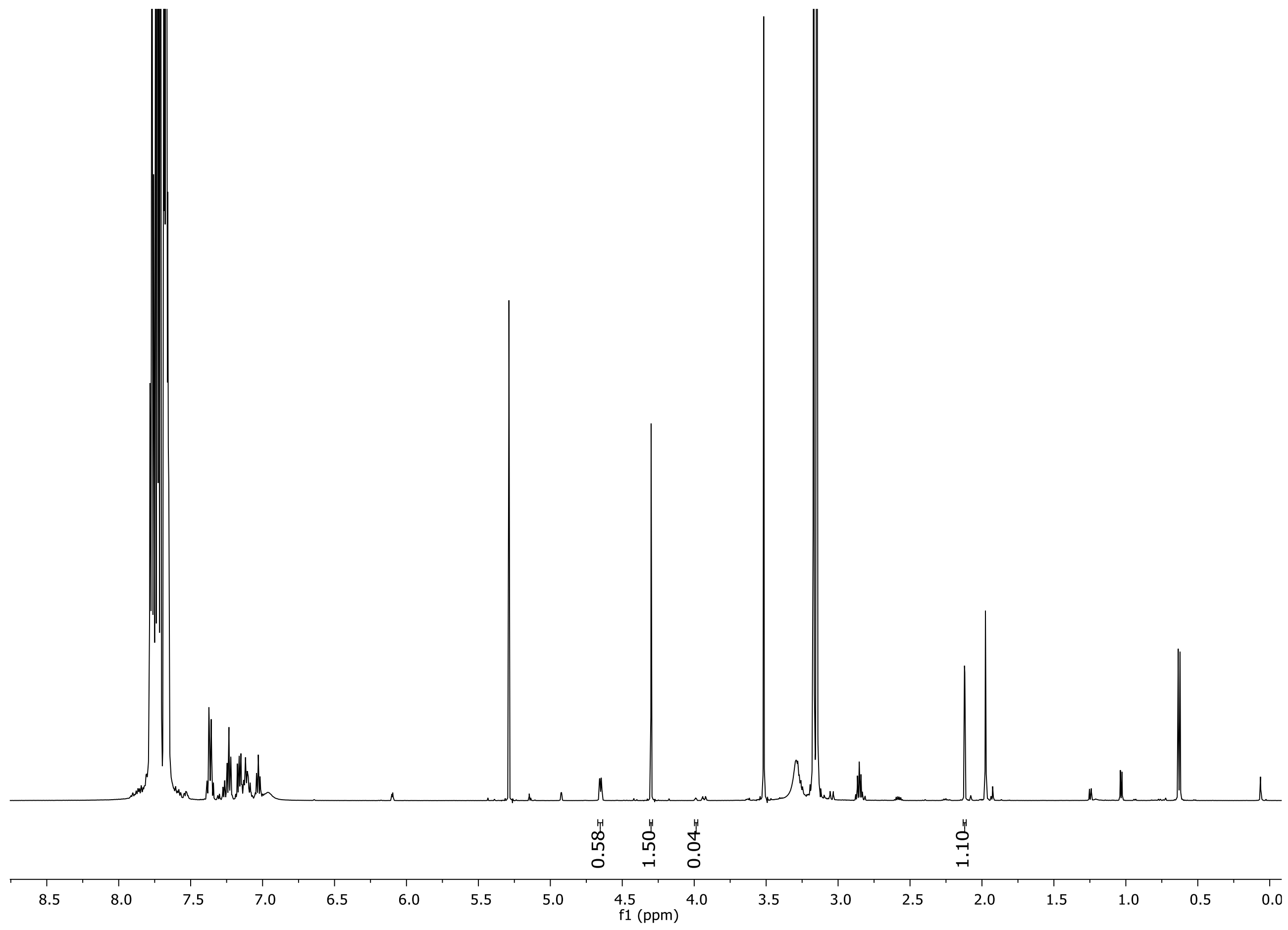

Figure S10. ^{1}H NMR of the experiment 3, Table 3.

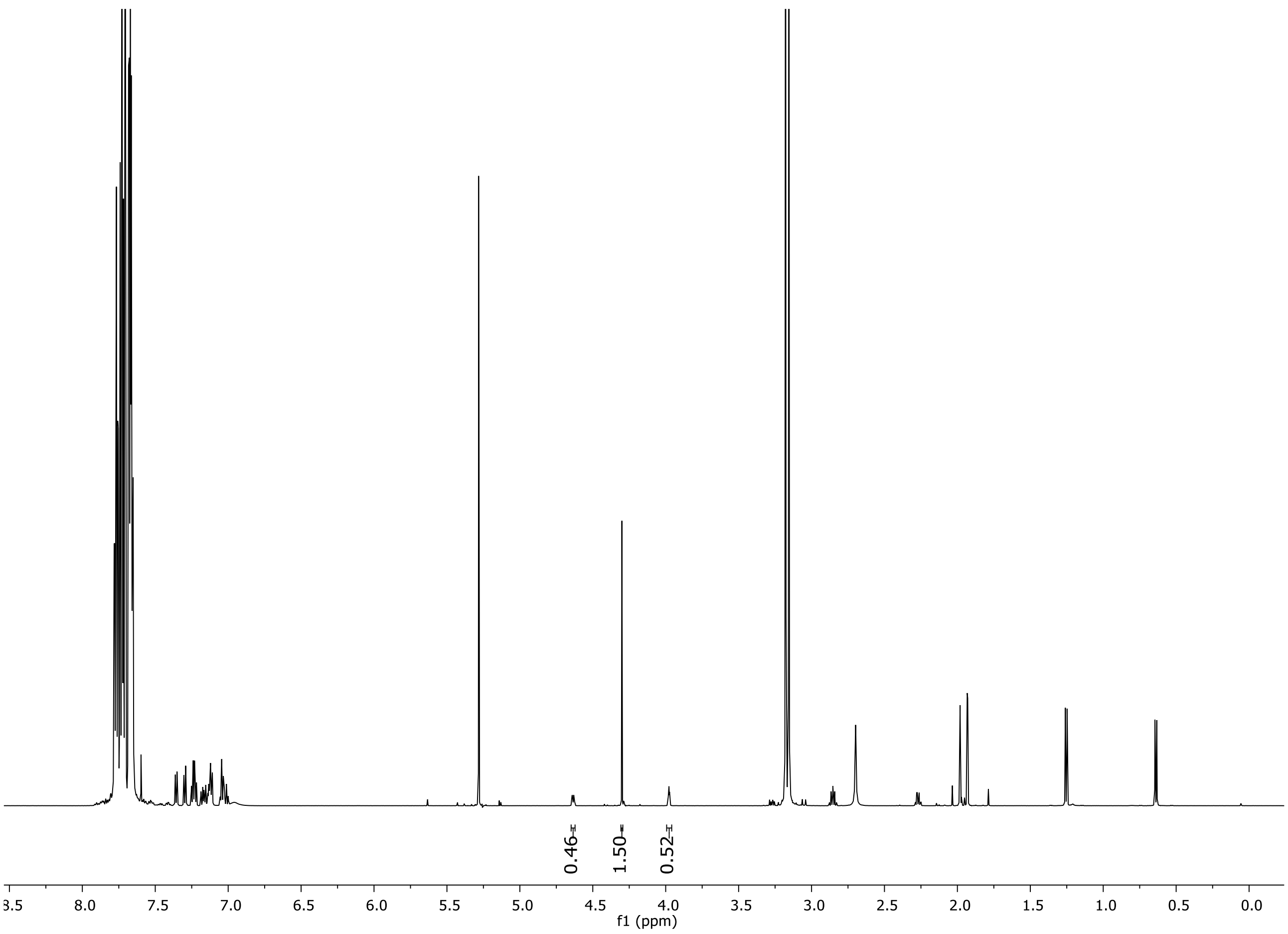

Figure S11. ^{1}H NMR of the experiment 4, Table 3.

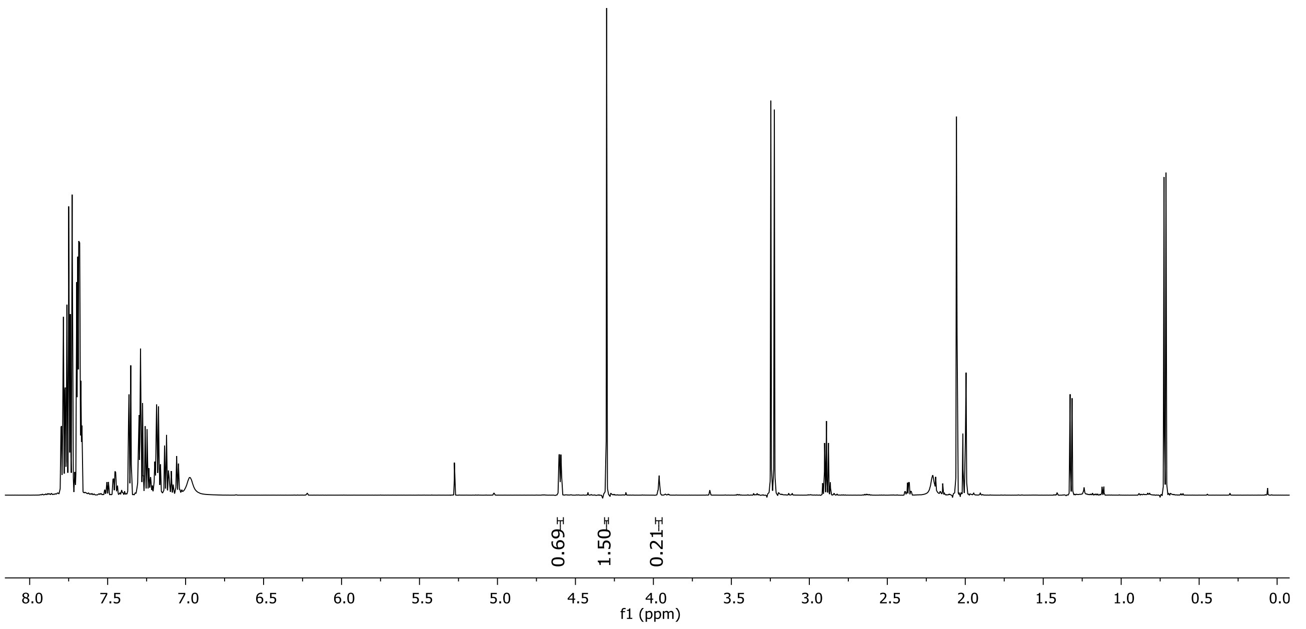

Figure S12. ^{1}H NMR of the experiment 5, Table 3.

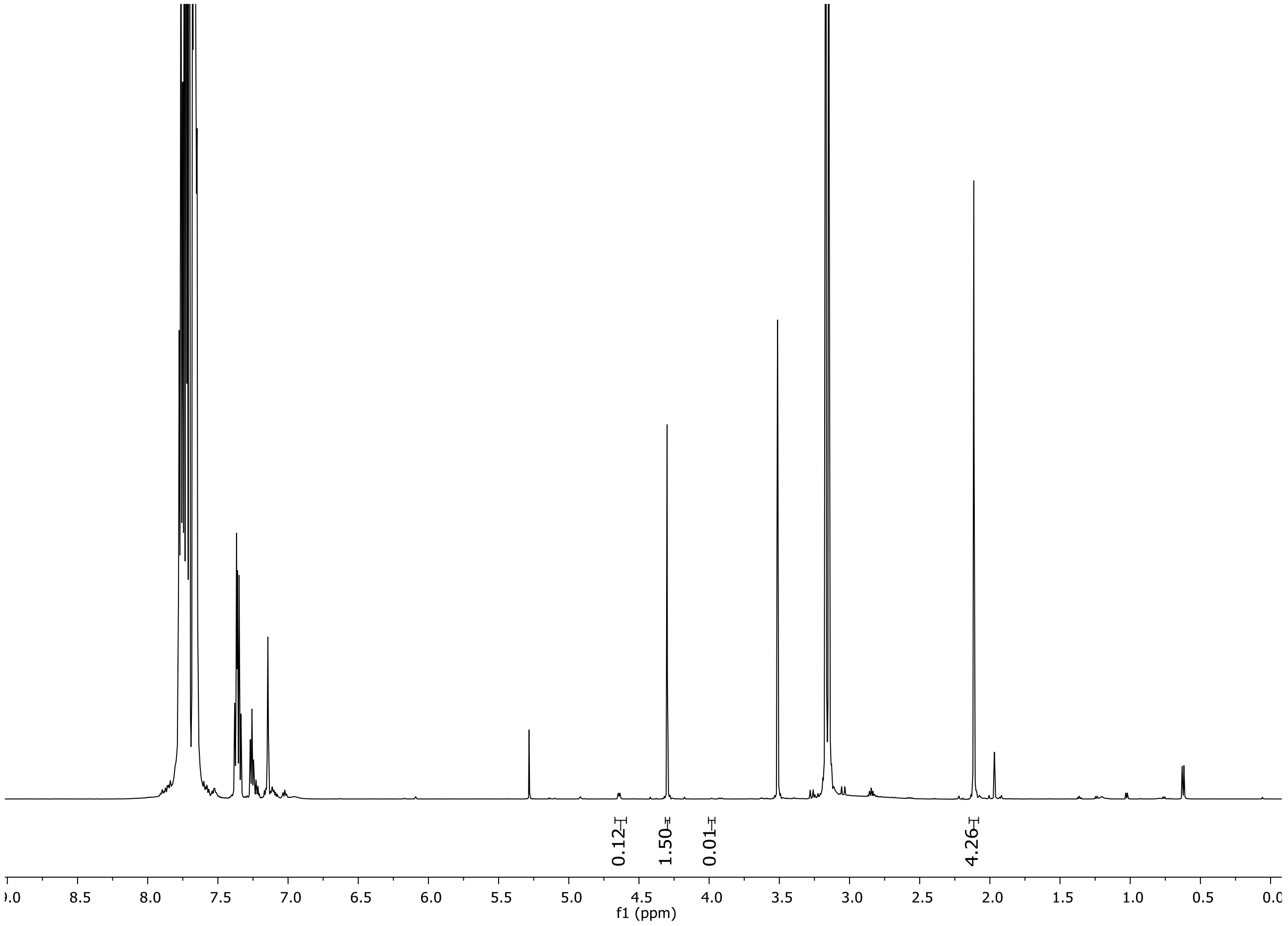

Figure S13. ^{1}H NMR of the experiment 6, Table 3.

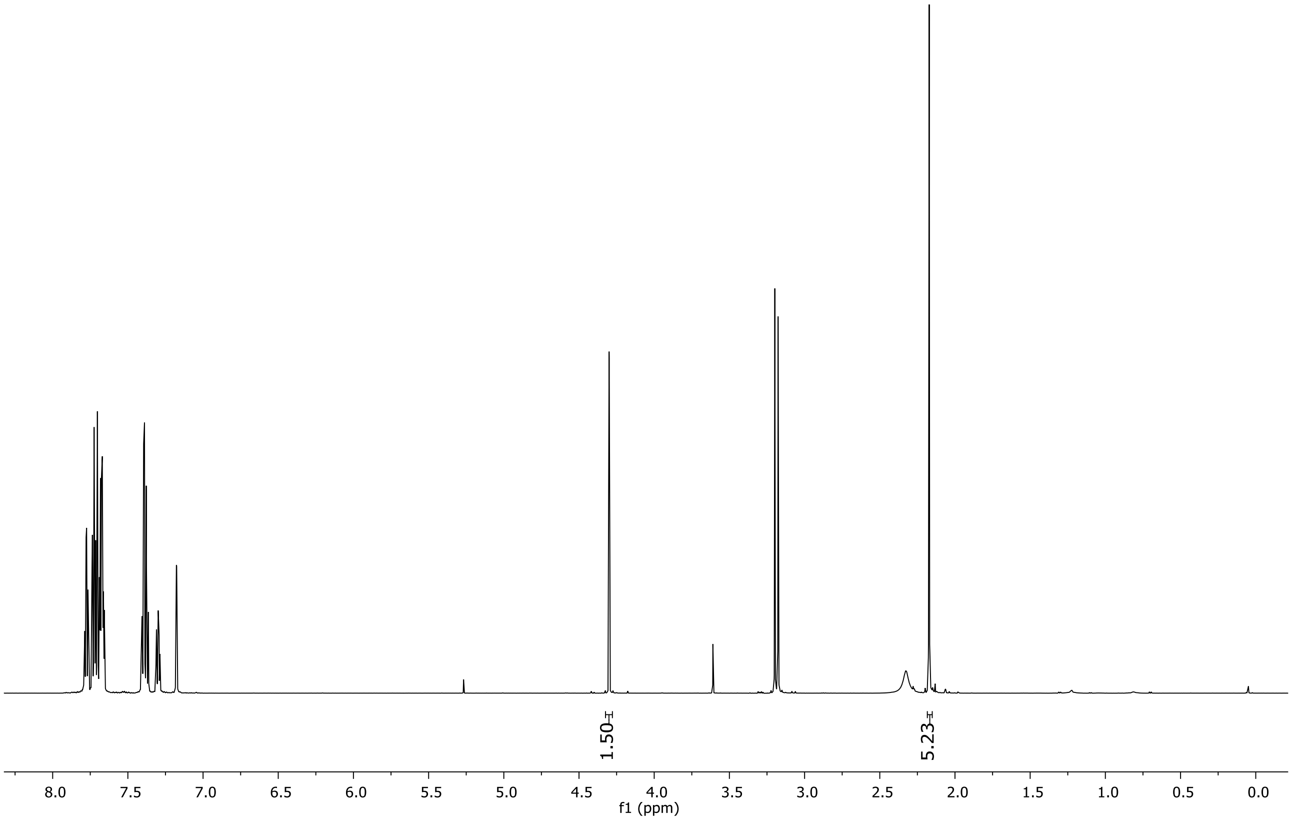

Figure S14. ^{1}H NMR of the experiment 7, Table 3.

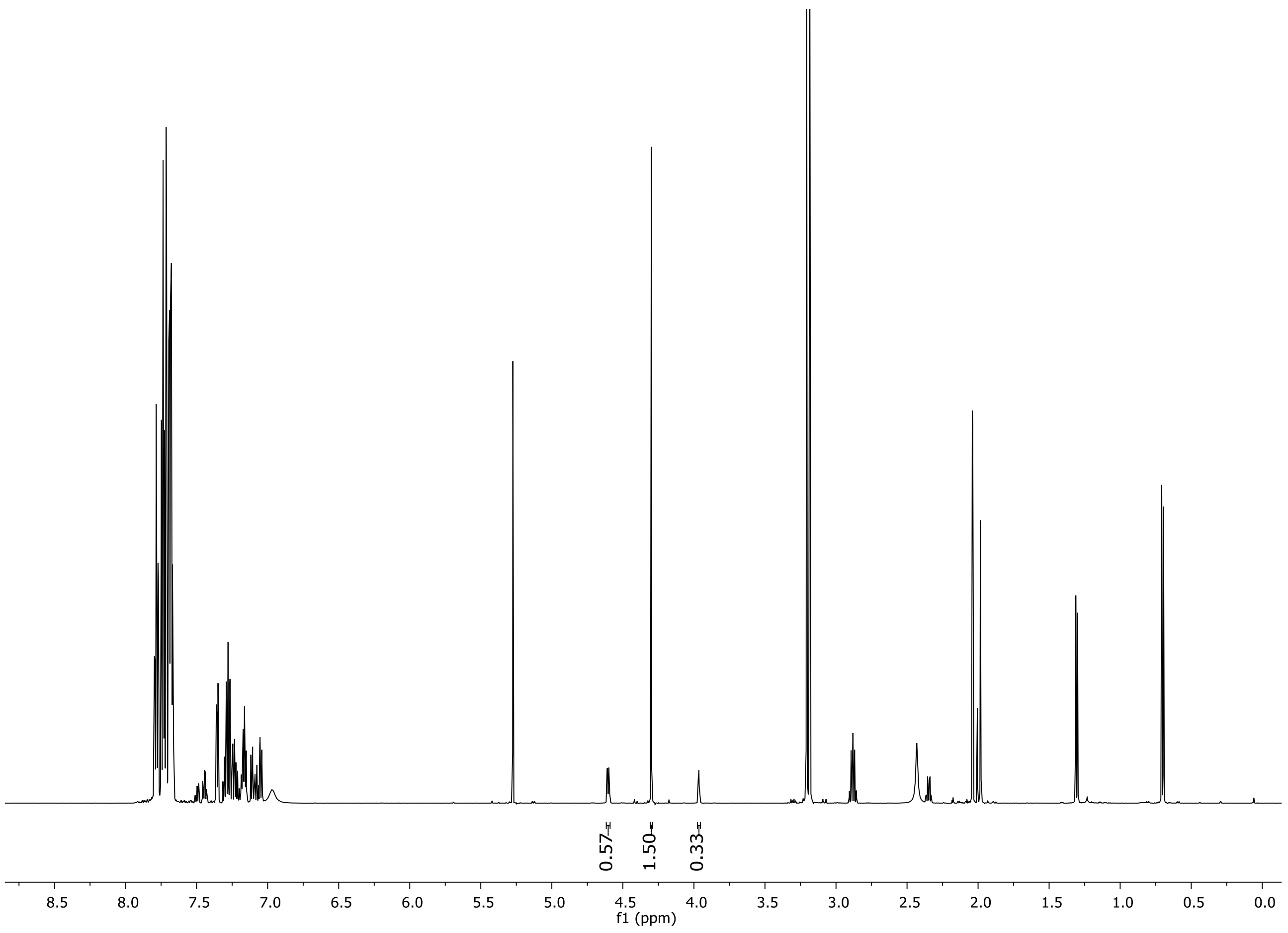

Figure S15. ^{1}H NMR of the experiment 8, Table 3.

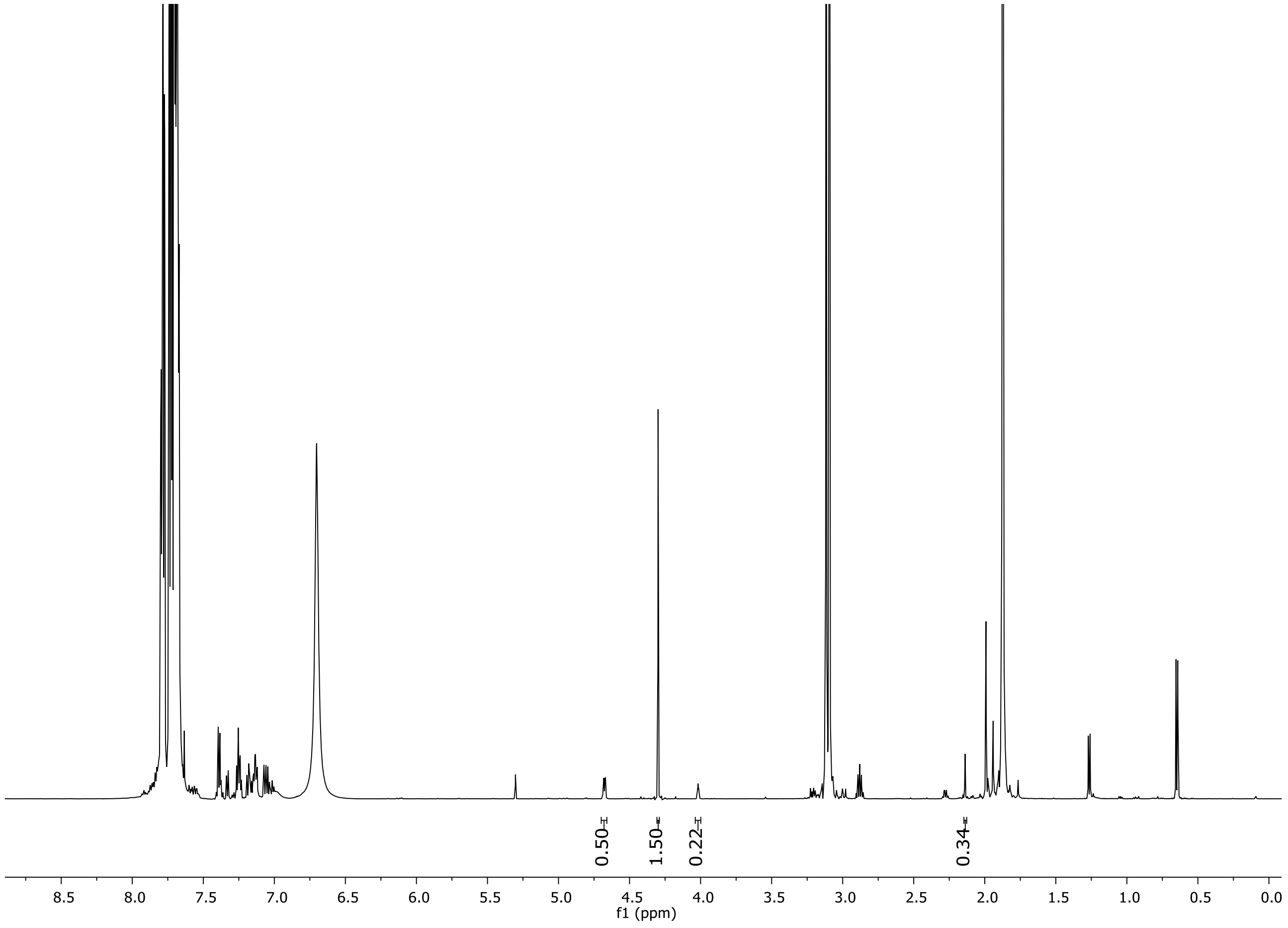

Figure S16. ^{1}H NMR of the experiment 9, Table 3.

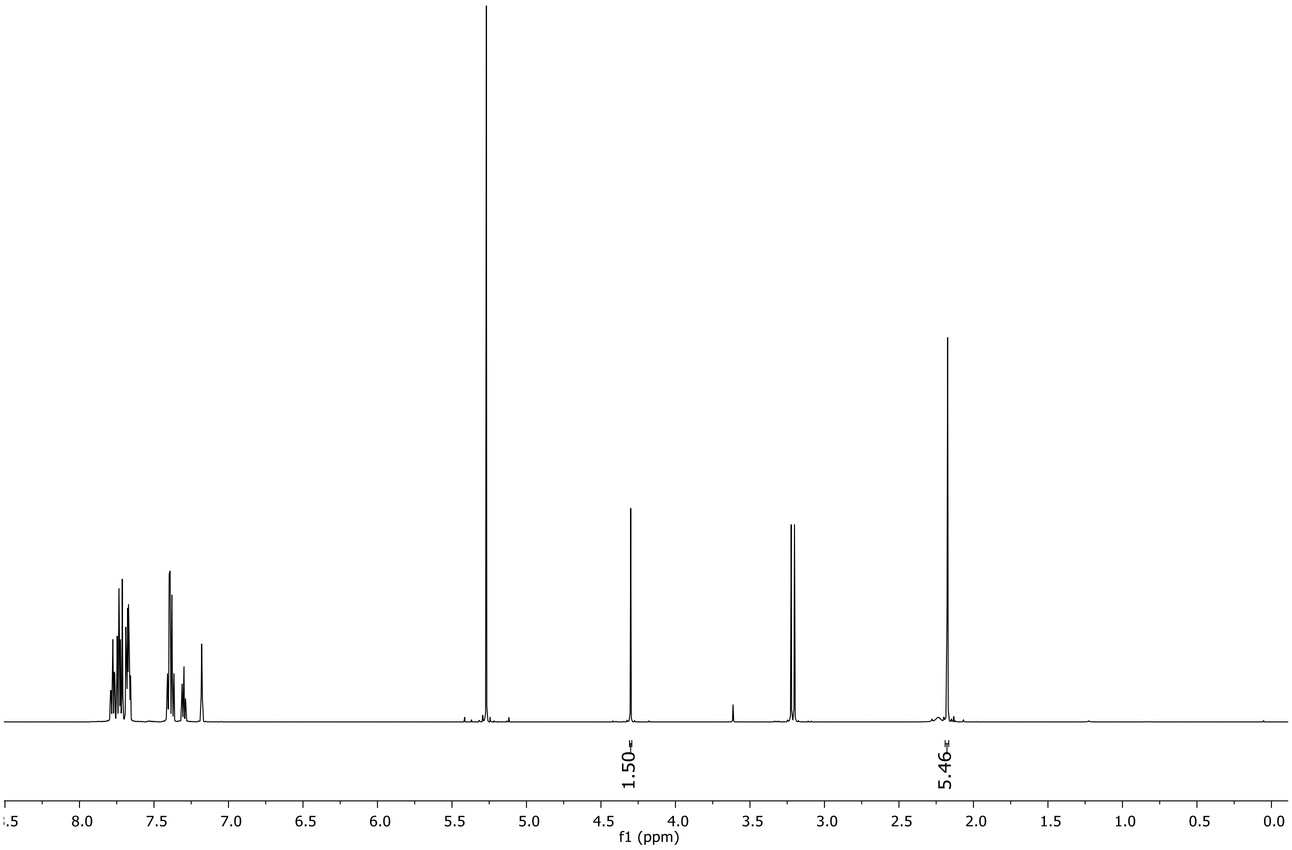

Figure S17. ^{1}H NMR of the experiment 10, Table 3.

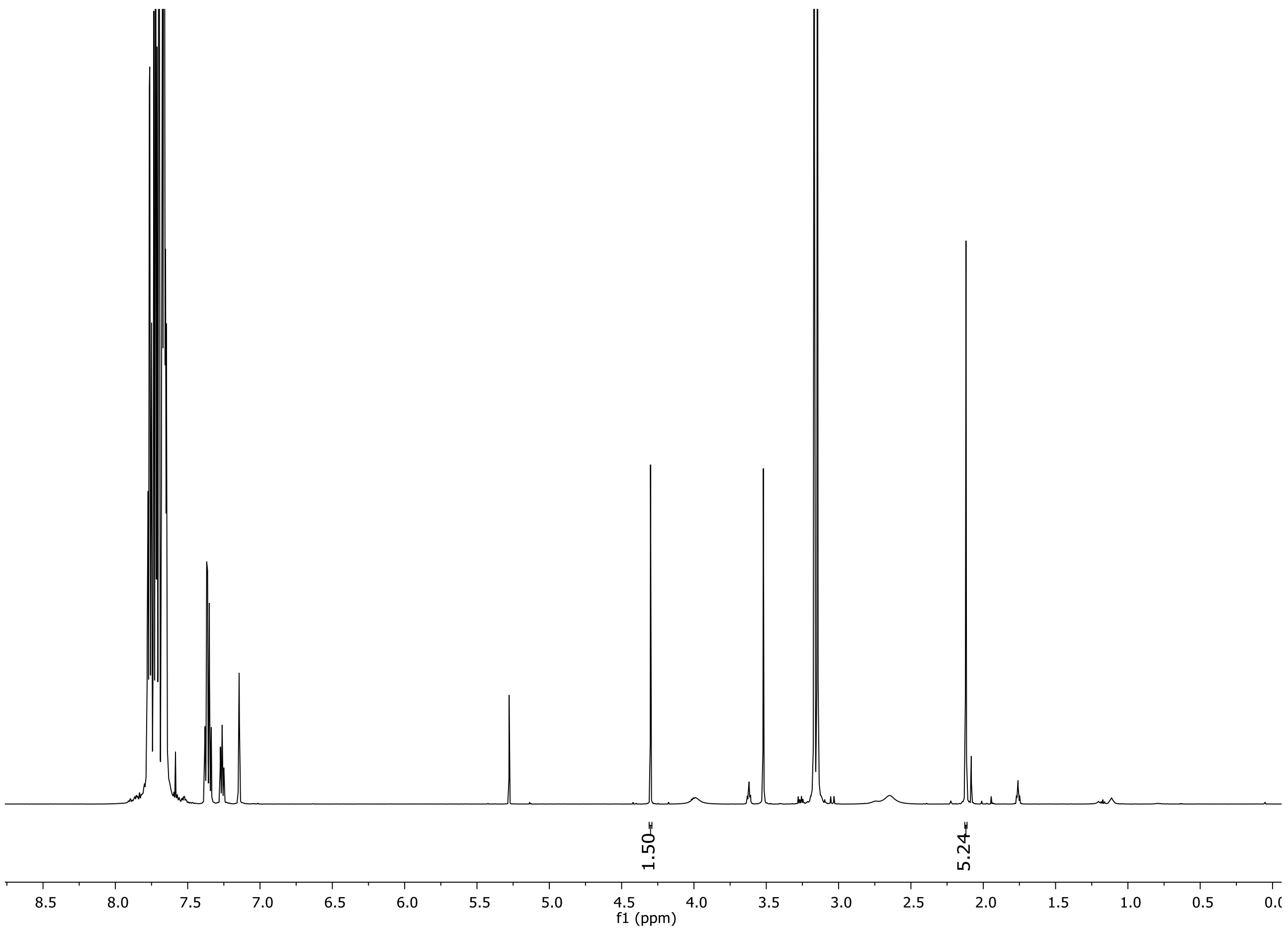

Figure S18. ^{1}H NMR of the experiment 11, Table 3.

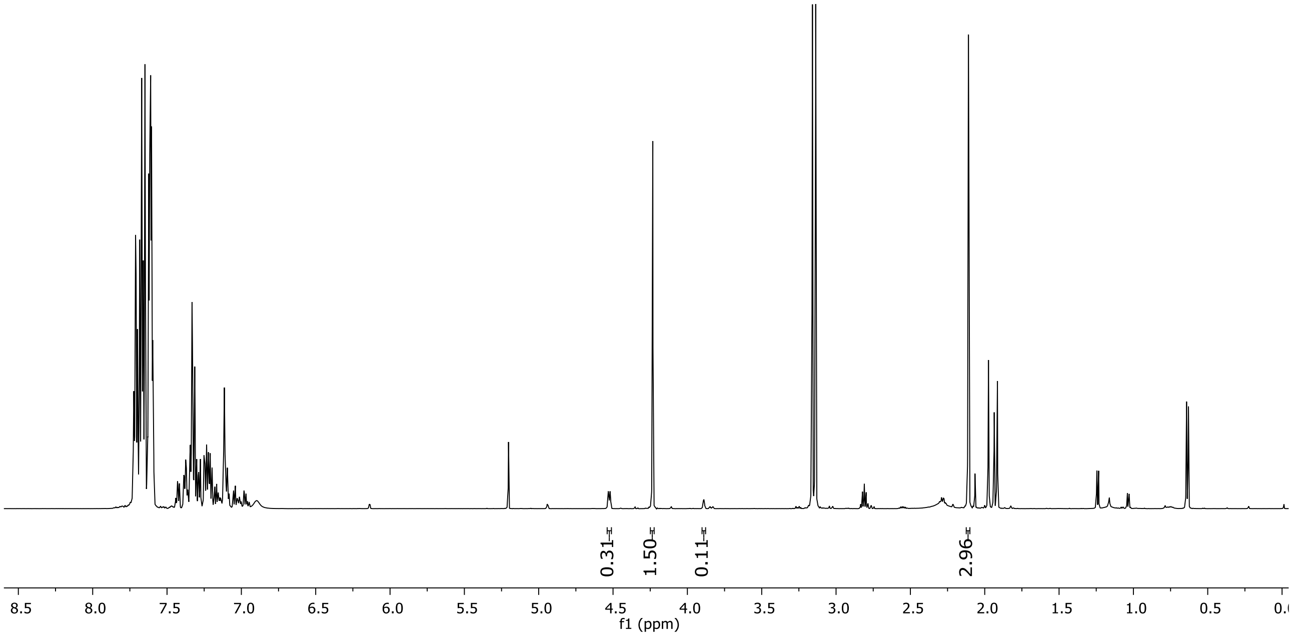

Figure S19. ^{1}H NMR of the experiment 12, Table 3.

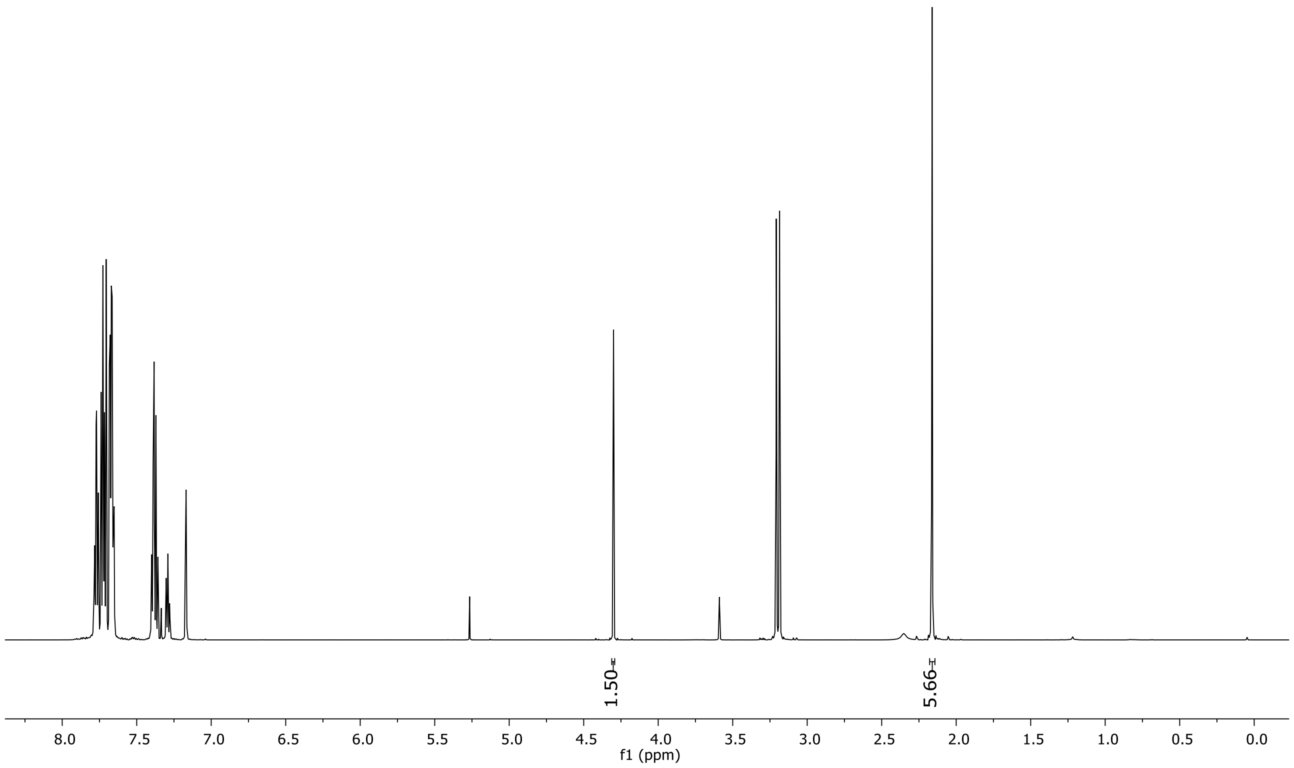

Figure S20. ^{1}H NMR of the experiment 13, Table 3.

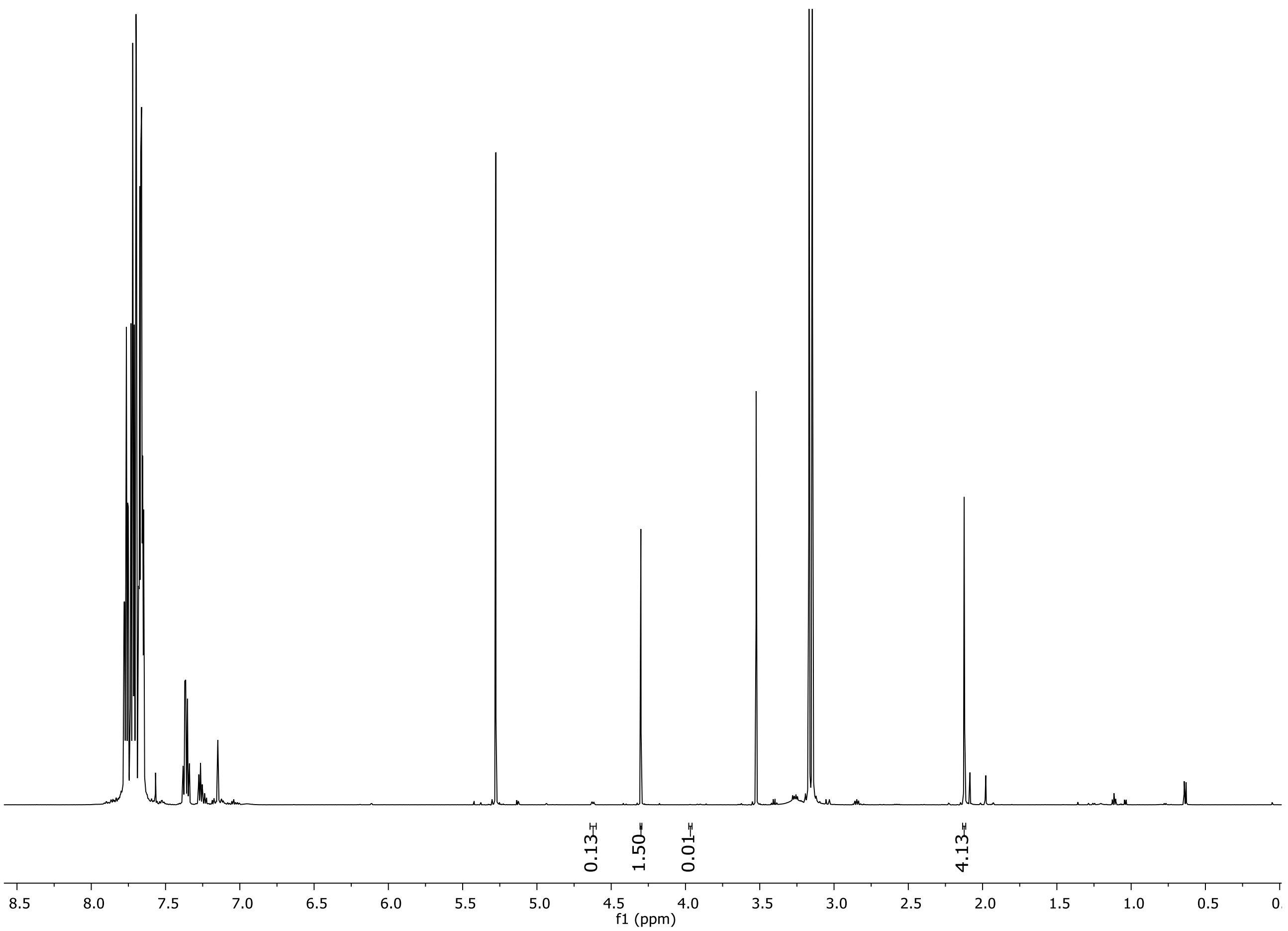

Figure S21. ^{1}H NMR of the experiment 14, Table 3.

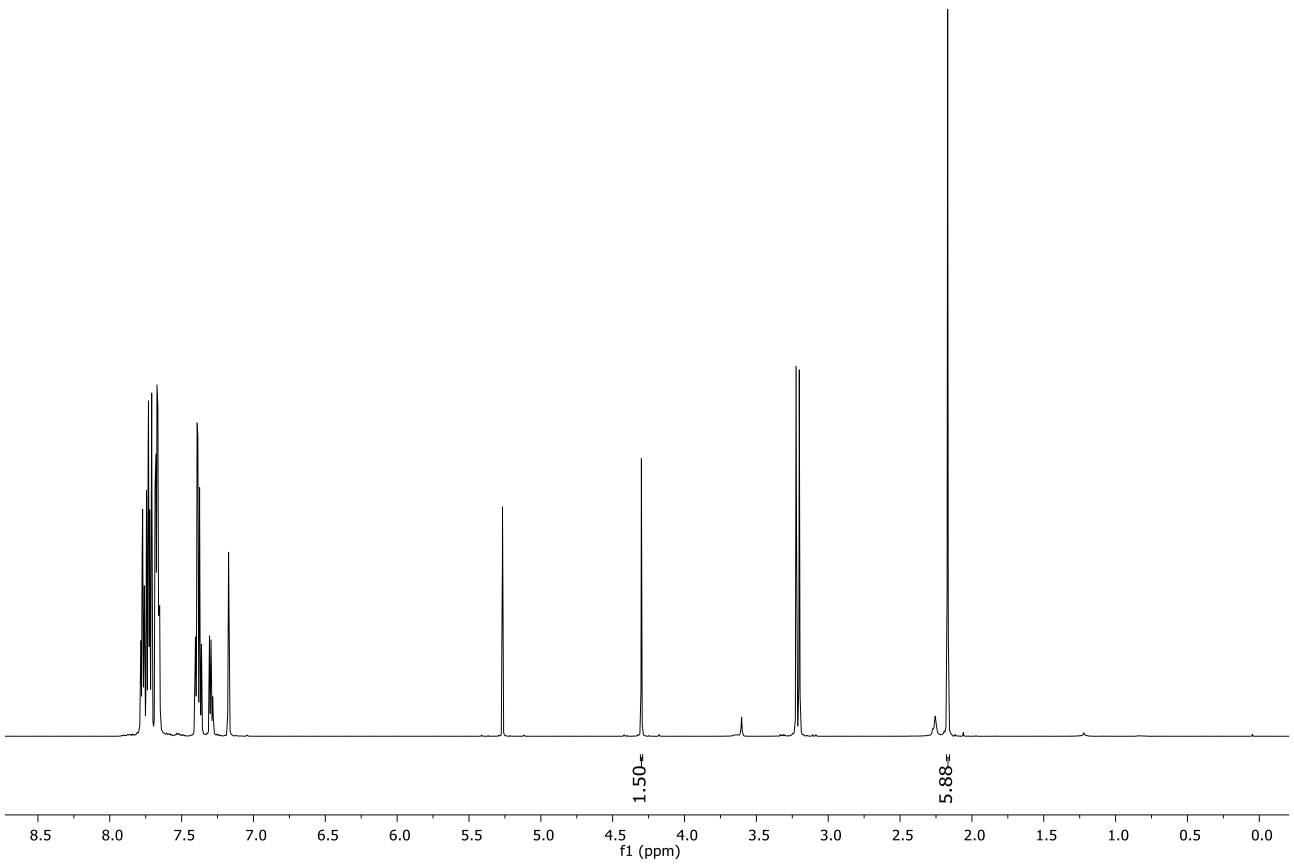

Figure S22. ^{1}H NMR of the experiment 15, Table 3.

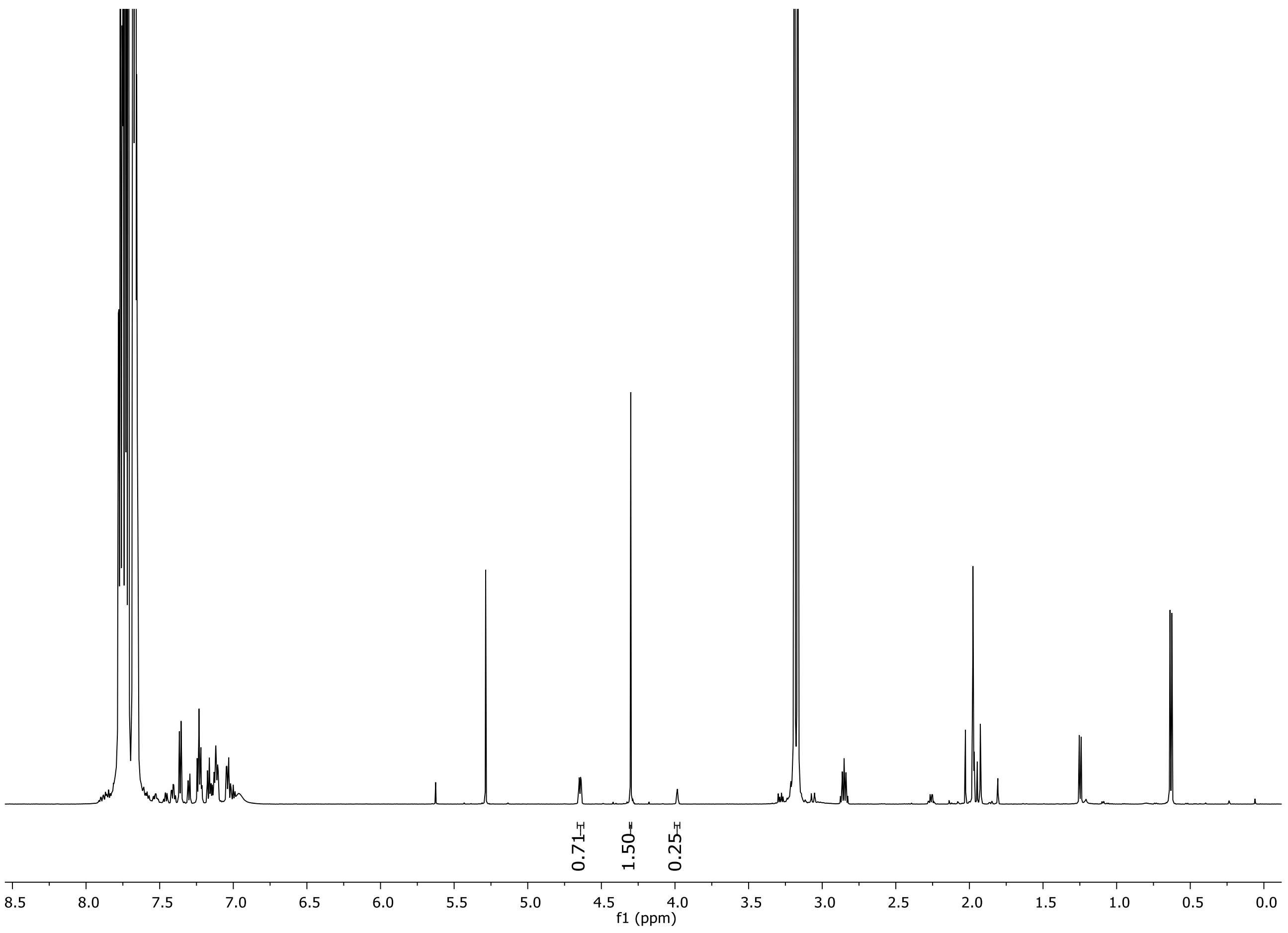

Figure S23. ^{1}H NMR of the experiment 16, Table 3.

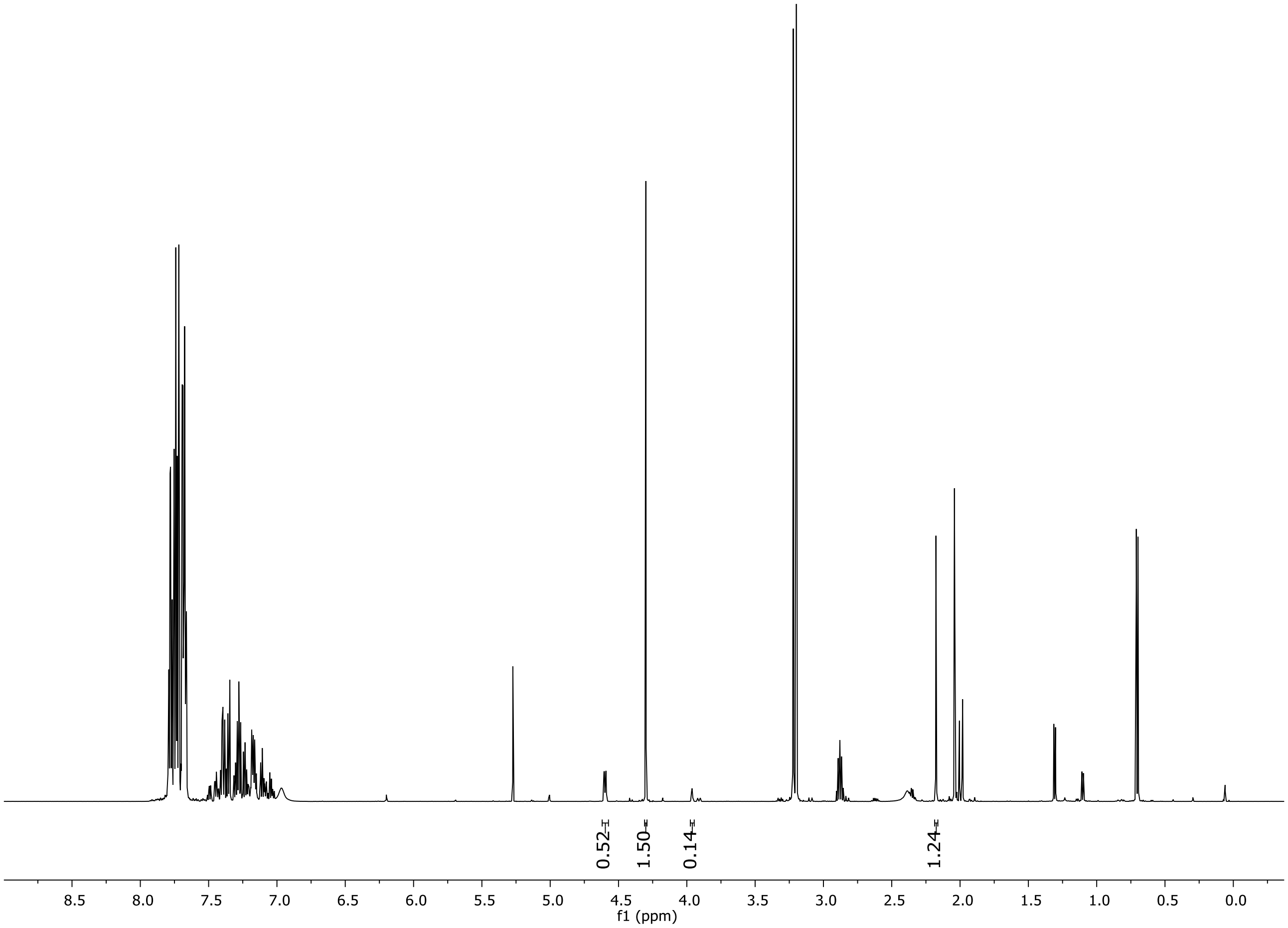

Figure S24. ^{1}H NMR of the experiment 1, Table 7.

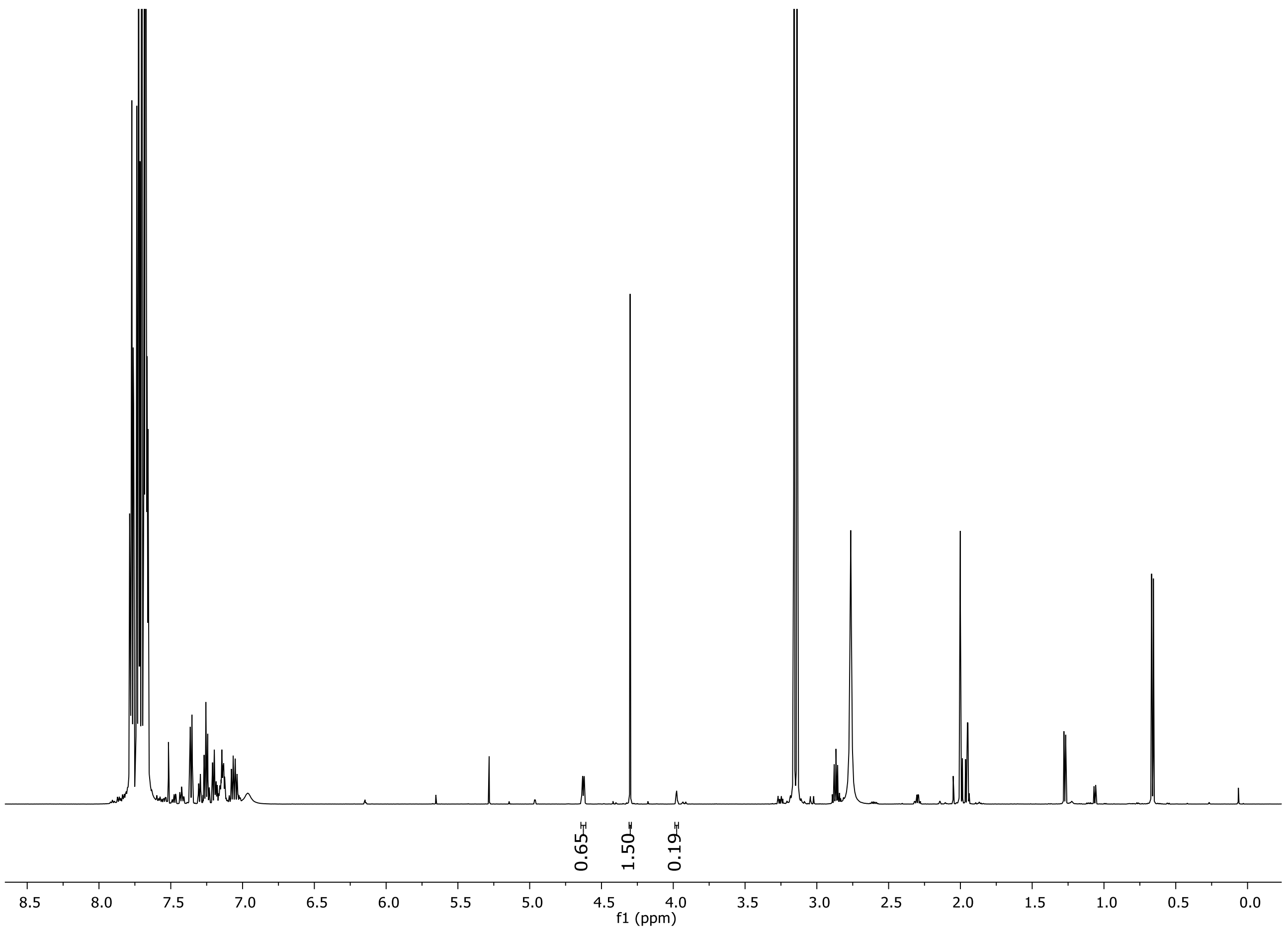

Figure S25. ^{1}H NMR of the experiment 2, Table 7.

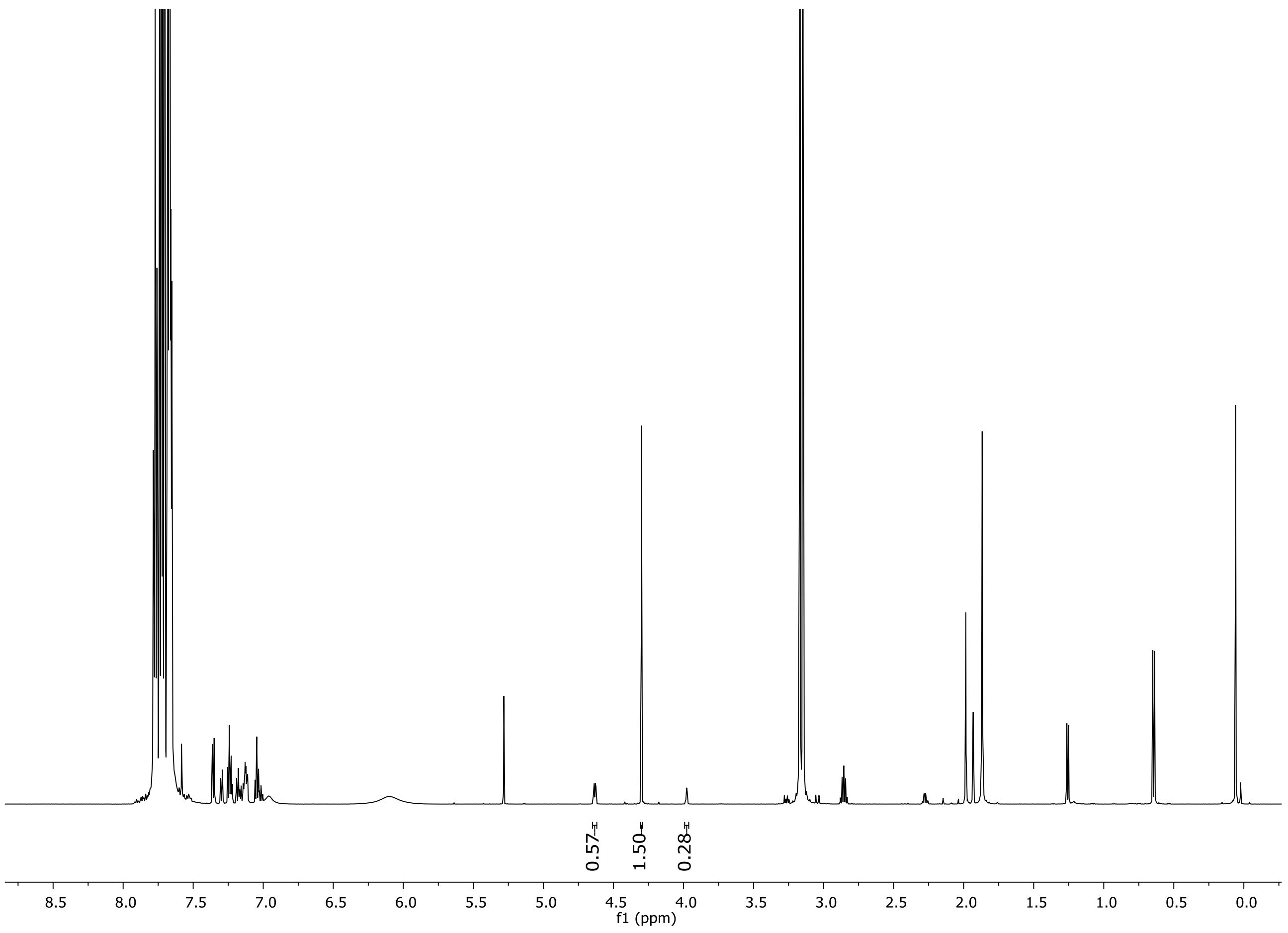

Figure S26. ^{1}H NMR of the experiment 3, Table 7.

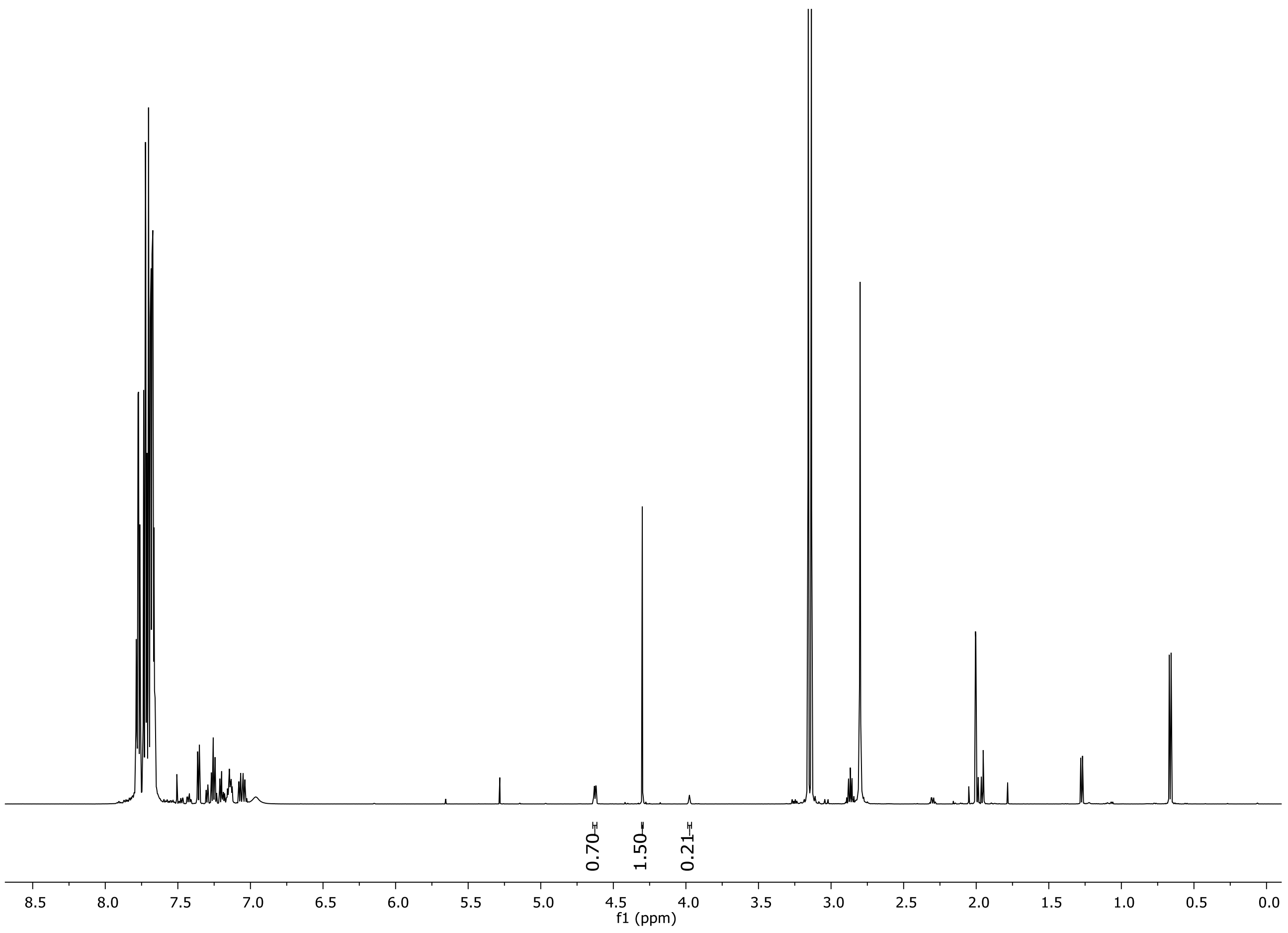

Figure S27. ^{1}H NMR of the experiment 4, Table 7.

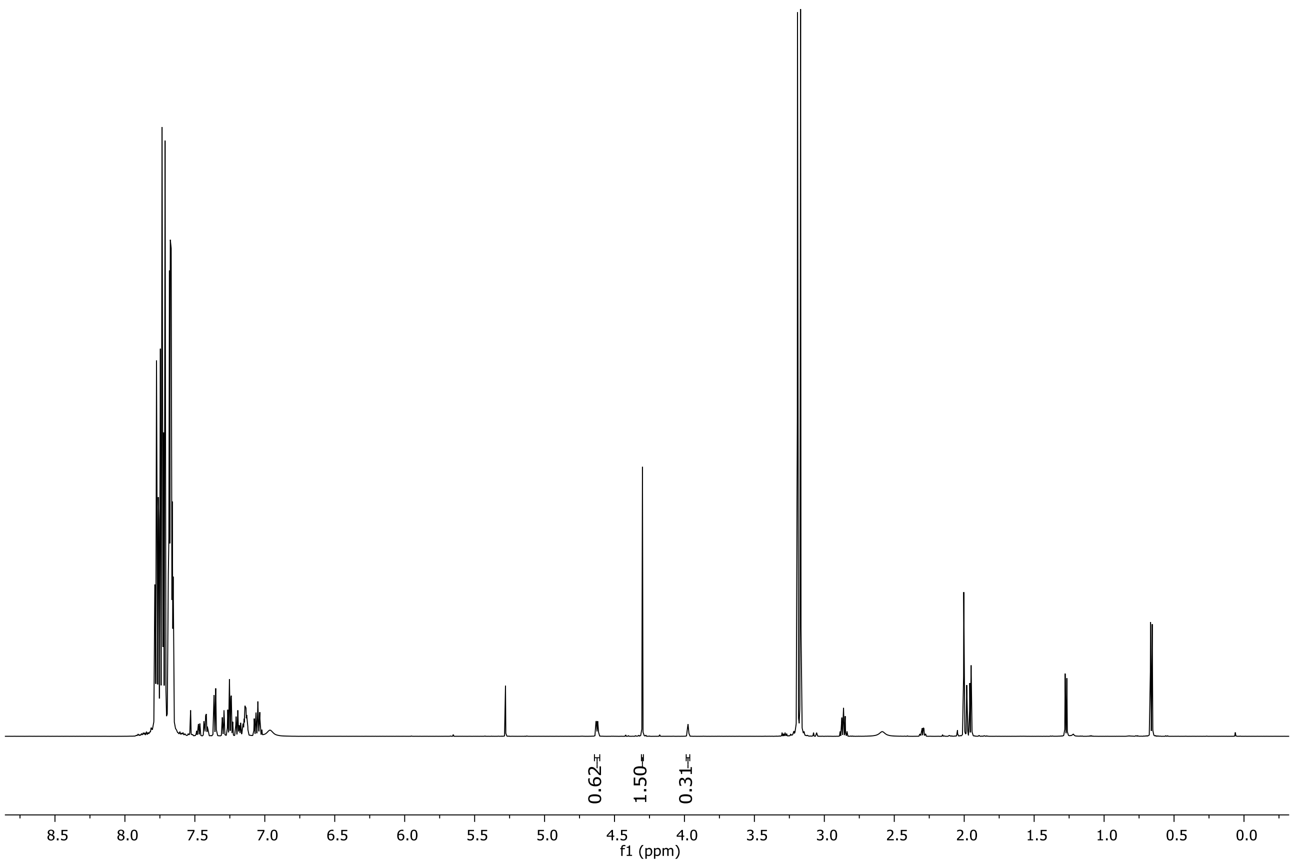

Figure S28. ^{1}H NMR of the experiment 5, Table 7.

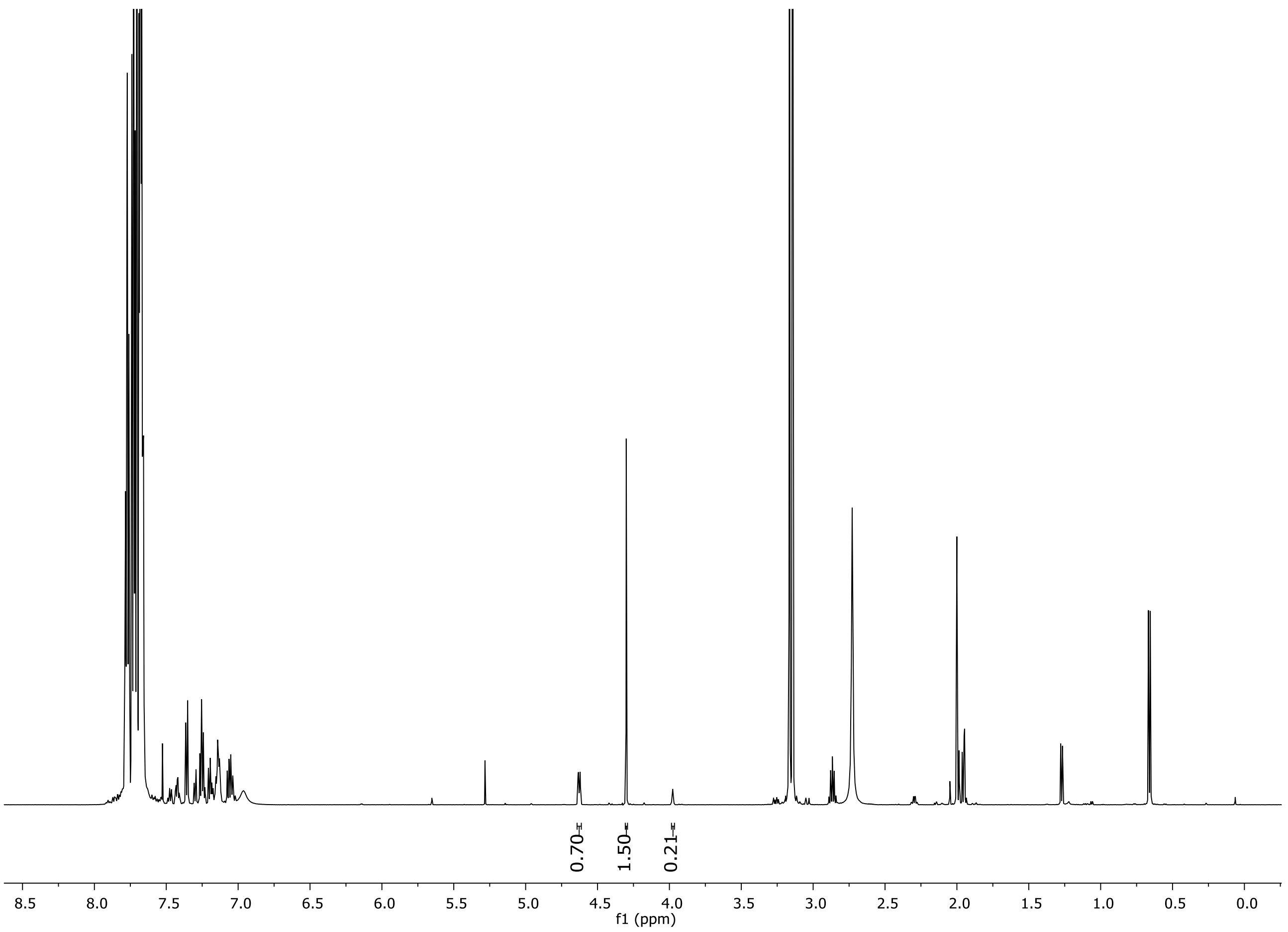

Figure S29. ^{1}H NMR of the experiment 6, Table 7.

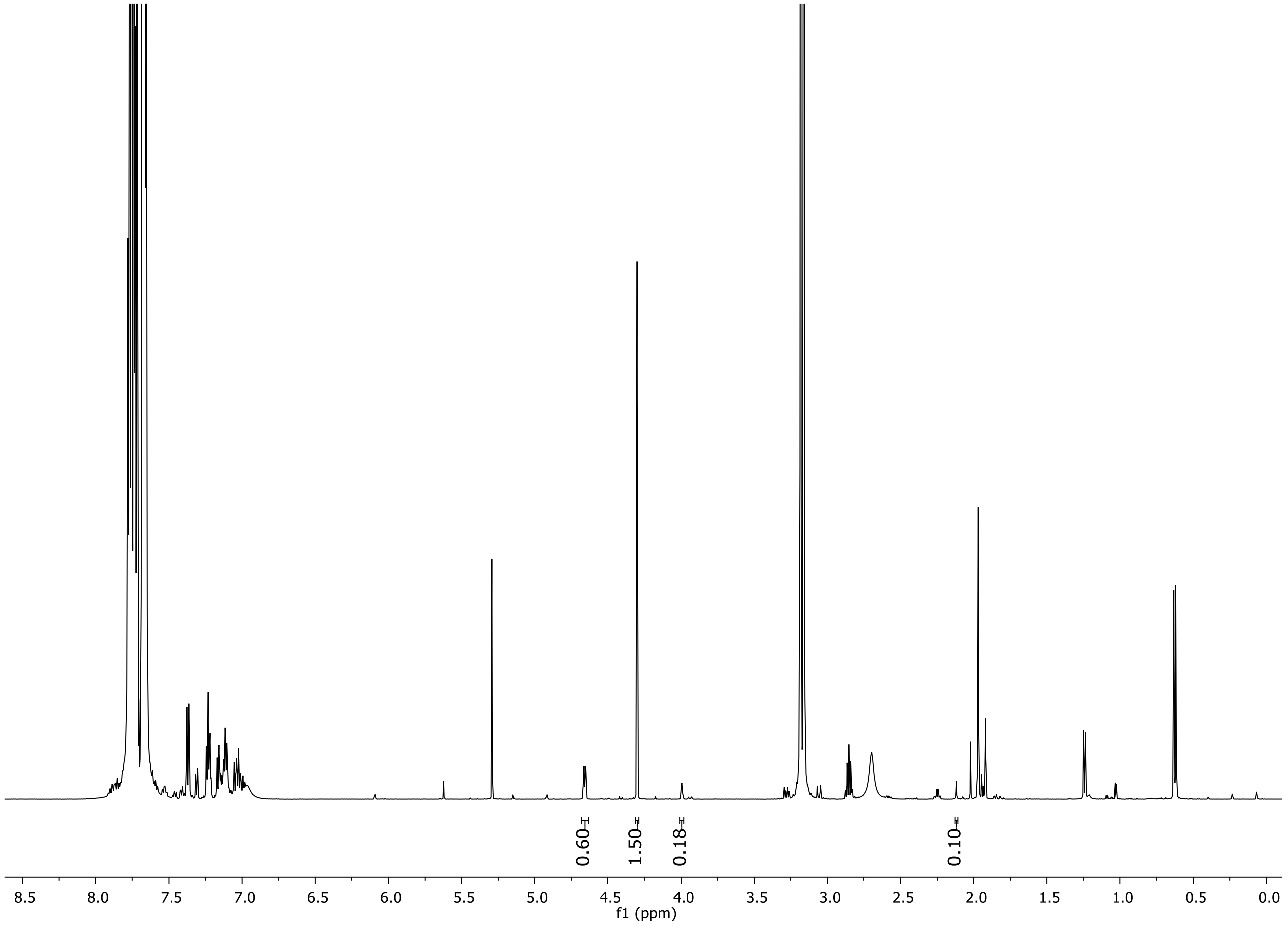

Figure S30. ^{1}H NMR of the experiment 7, Table 7.

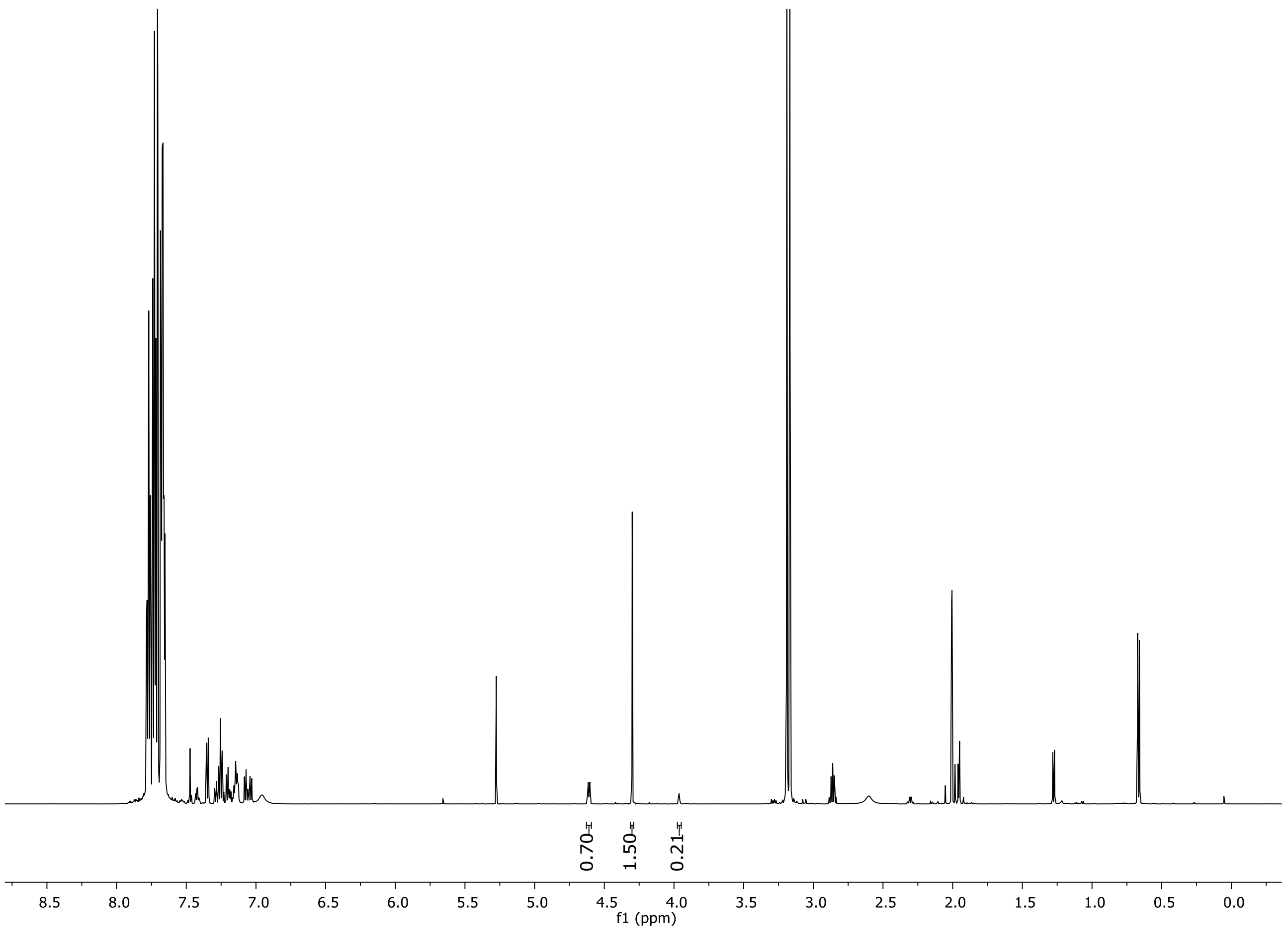

Figure S31. ^{1}H NMR of the experiment 8, Table 7.

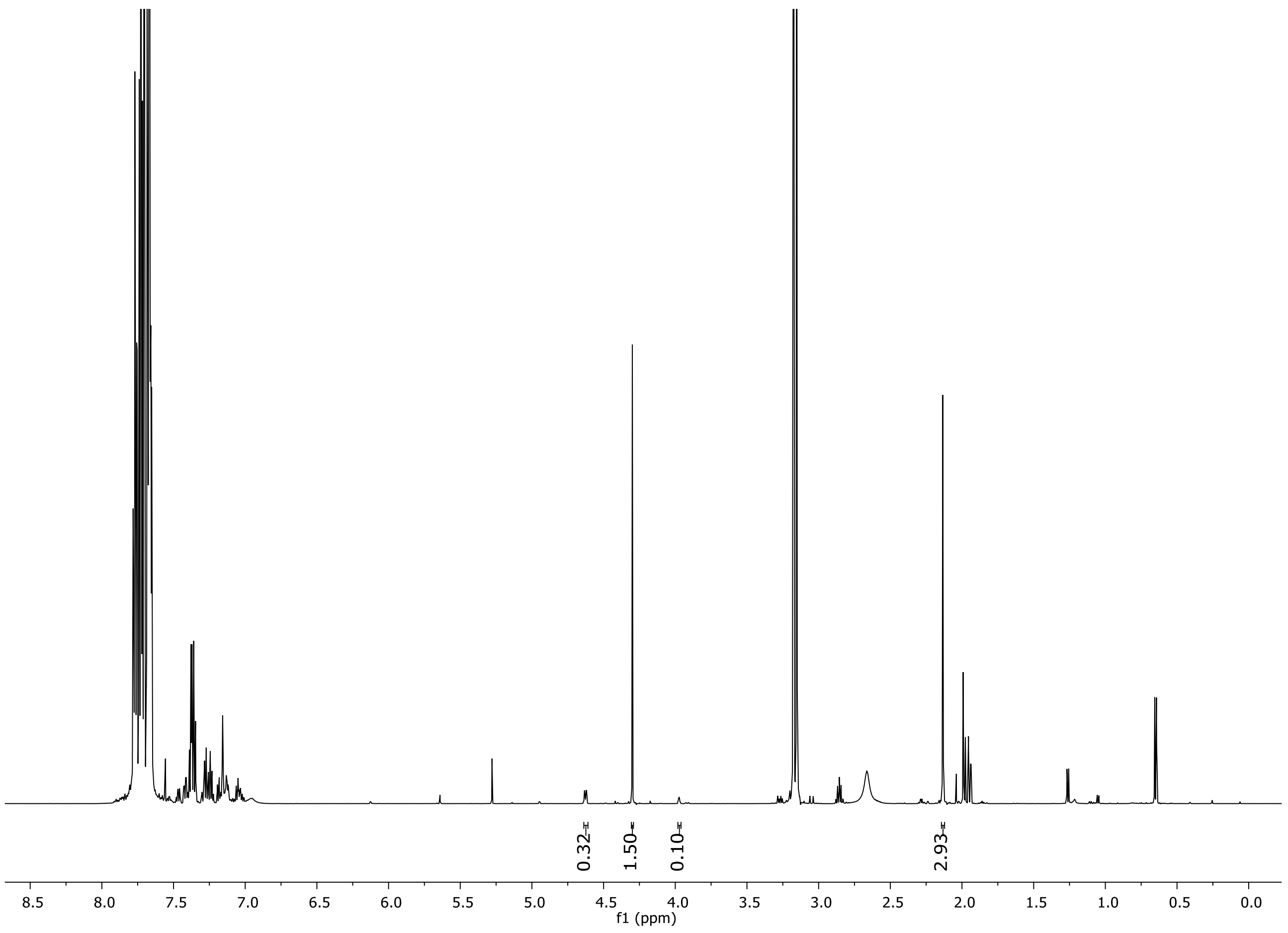

Figure S32. ^{1}H NMR of the experiment 9, Table 7.

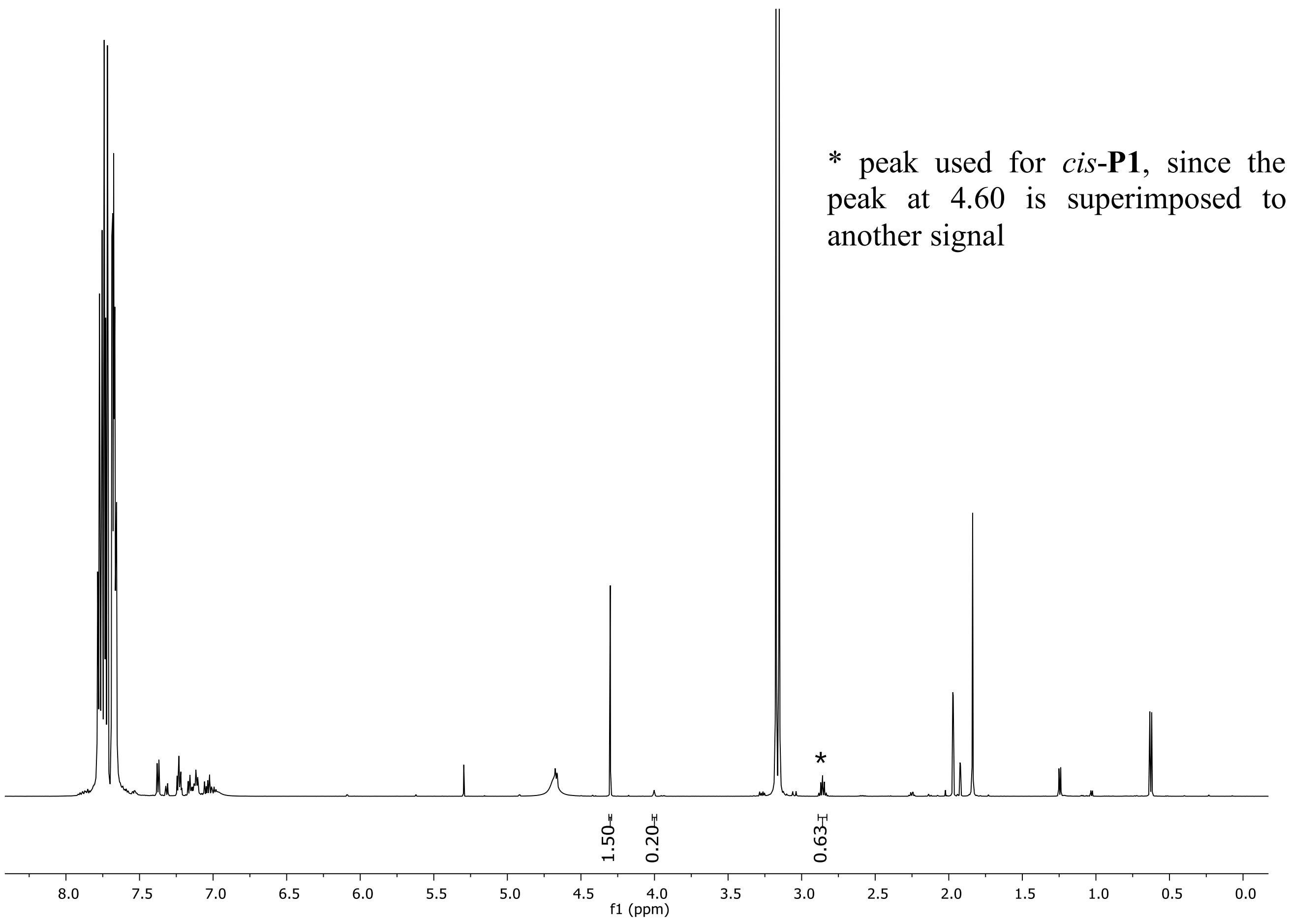

Figure S33. ^{1}H NMR of the experiment 10, Table 7.

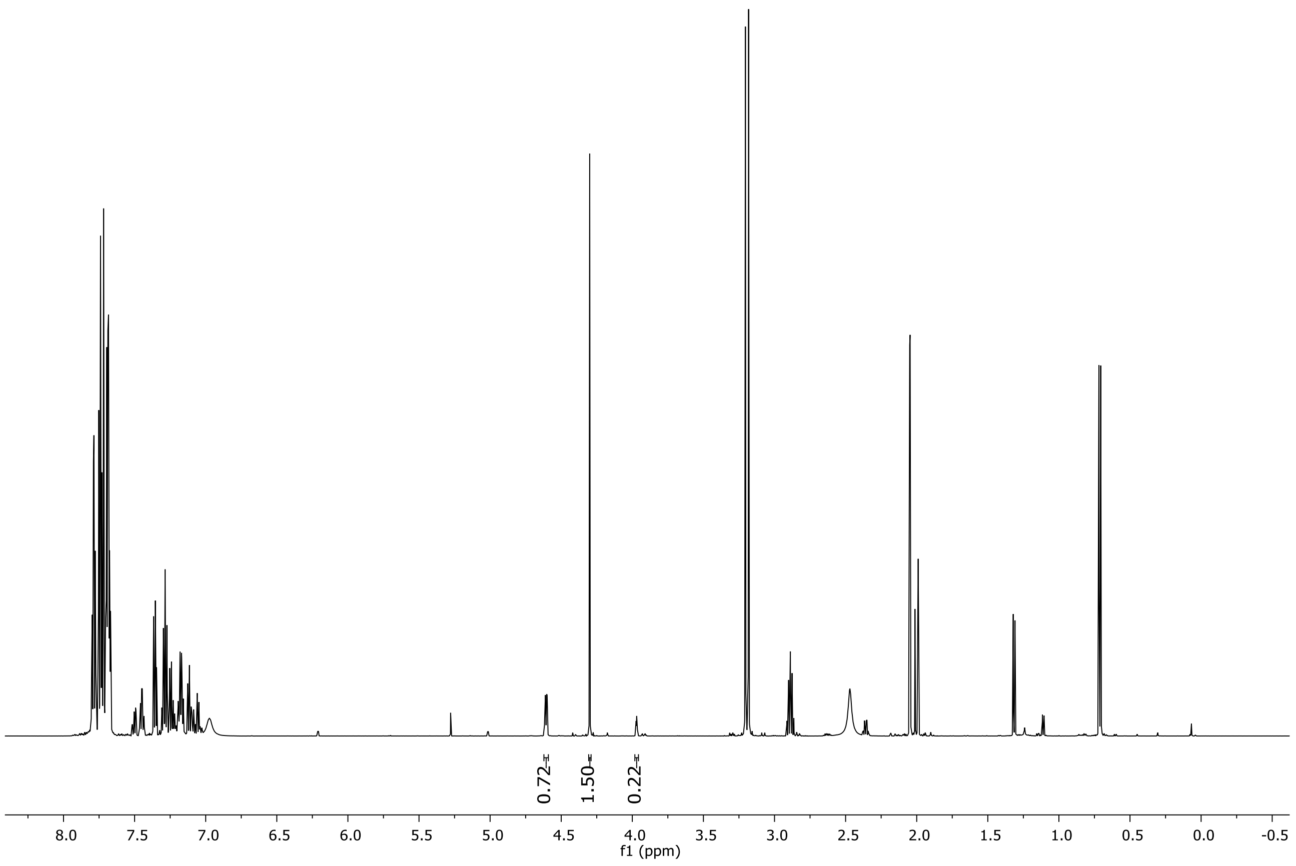

Figure S34. ^{1}H NMR of the experiment 11, Table 7.

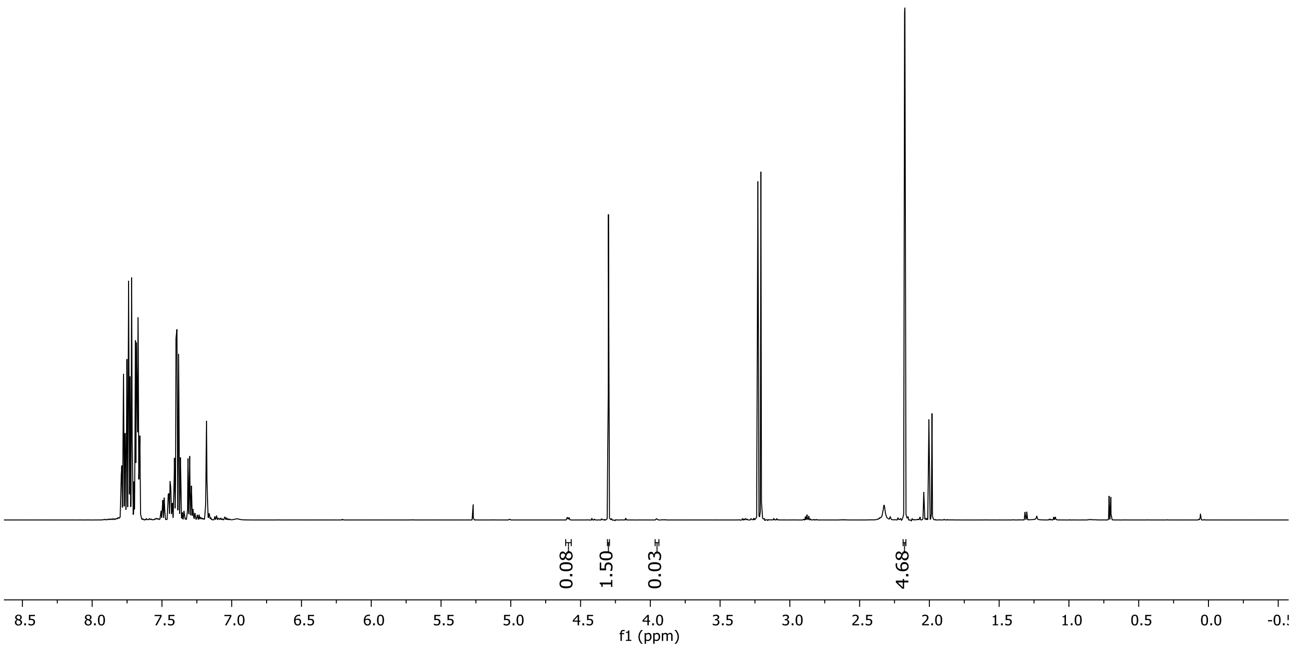

Figure S35. ^{1}H NMR of the experiment 12, Table 7.

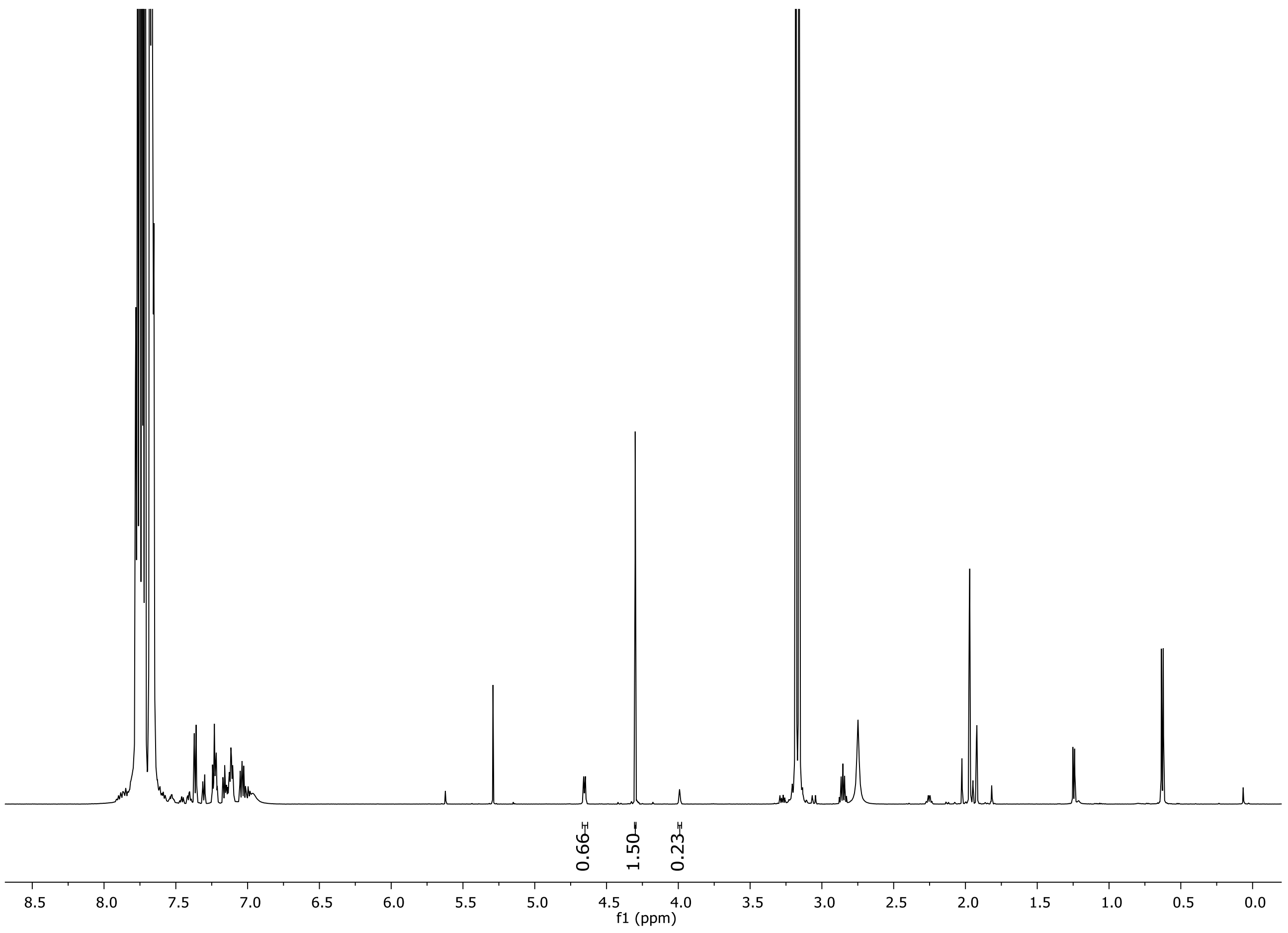

Figure S36. ^{1}H NMR of the experiment 13, Table 7.

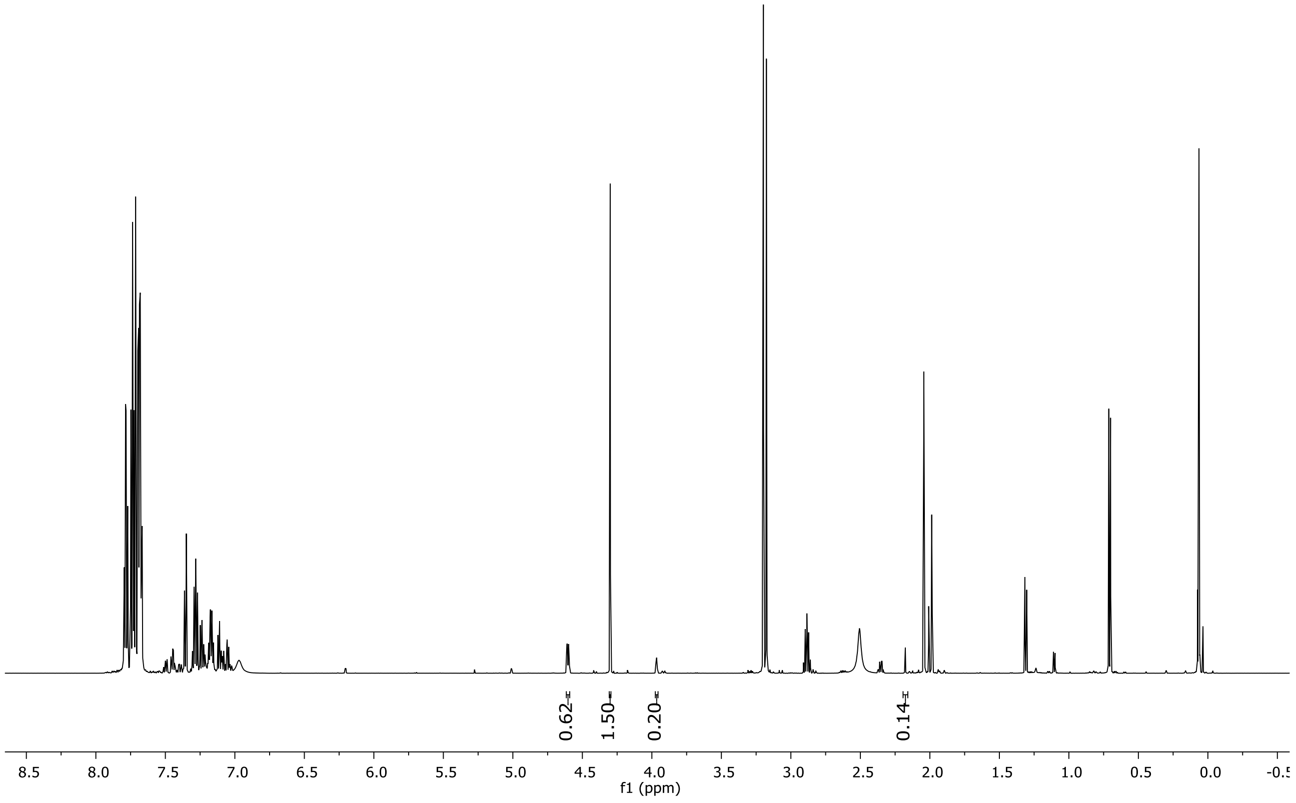

Figure S37. ^{1}H NMR of the experiment 14, Table 7.

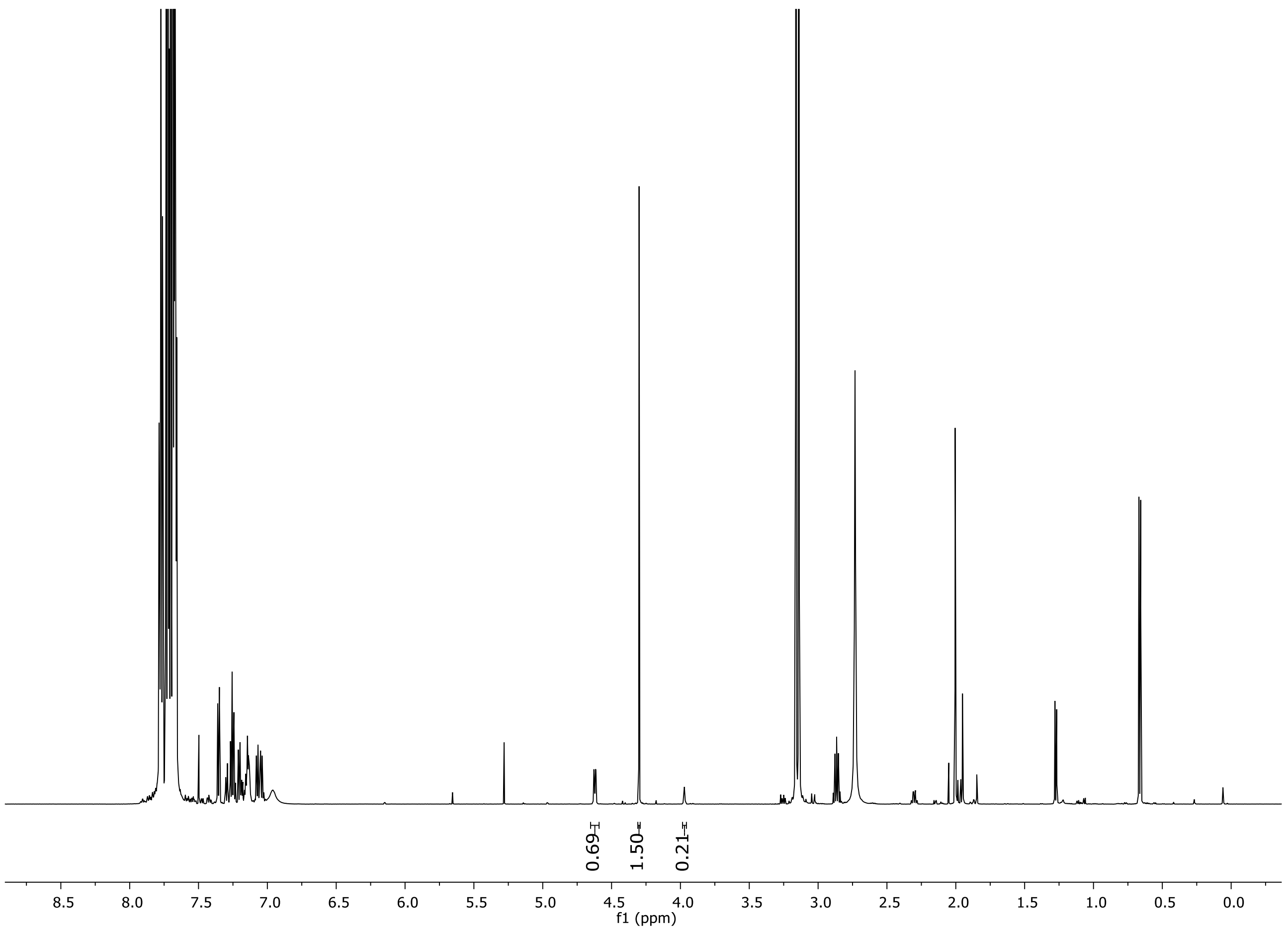

Figure S38. ^{1}H NMR of the experiment 15, Table 7.

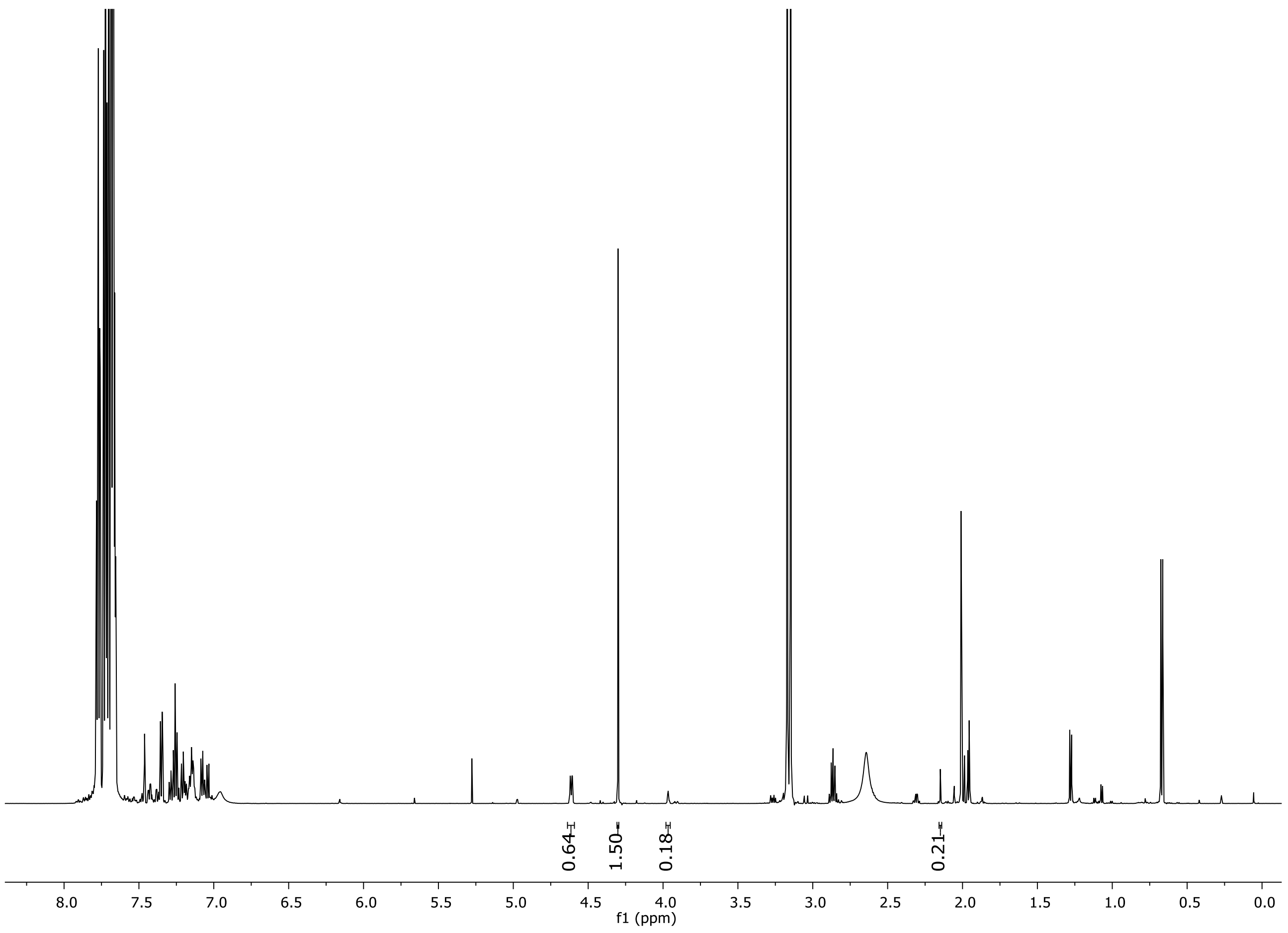

Figure S39. ^{1}H NMR of the experiment in Table 12.

Surface Responding Analysis

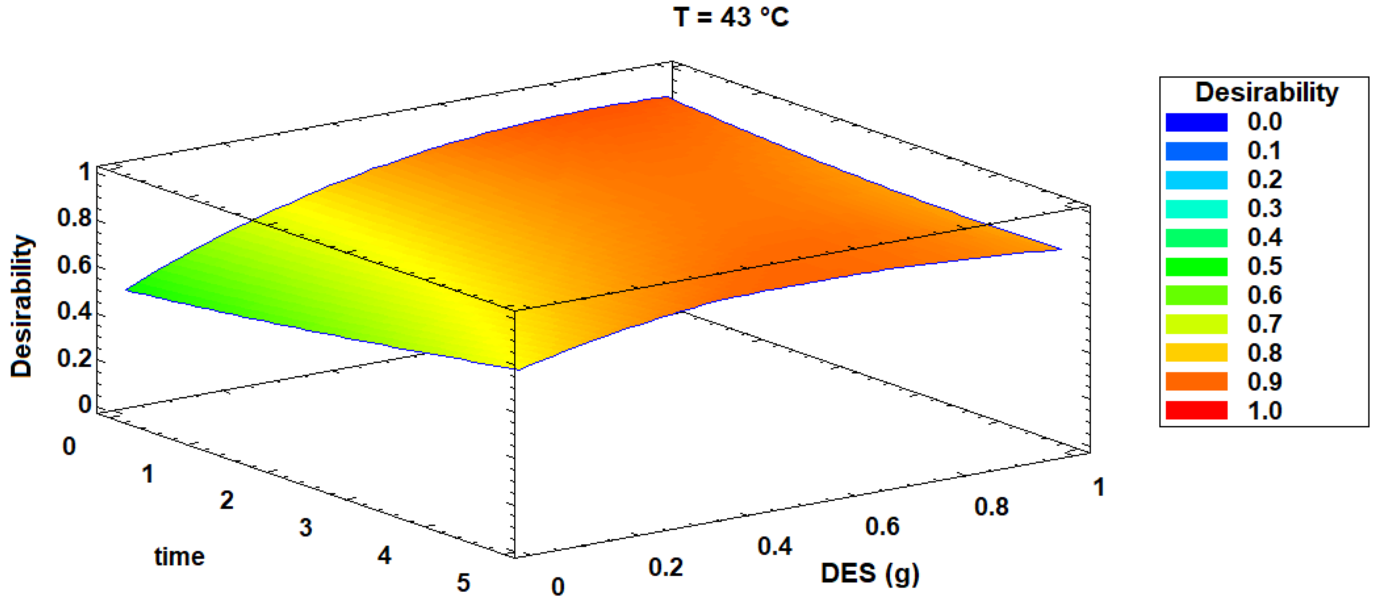

Figure S40. Surface Responding Analysis at 43 °C.

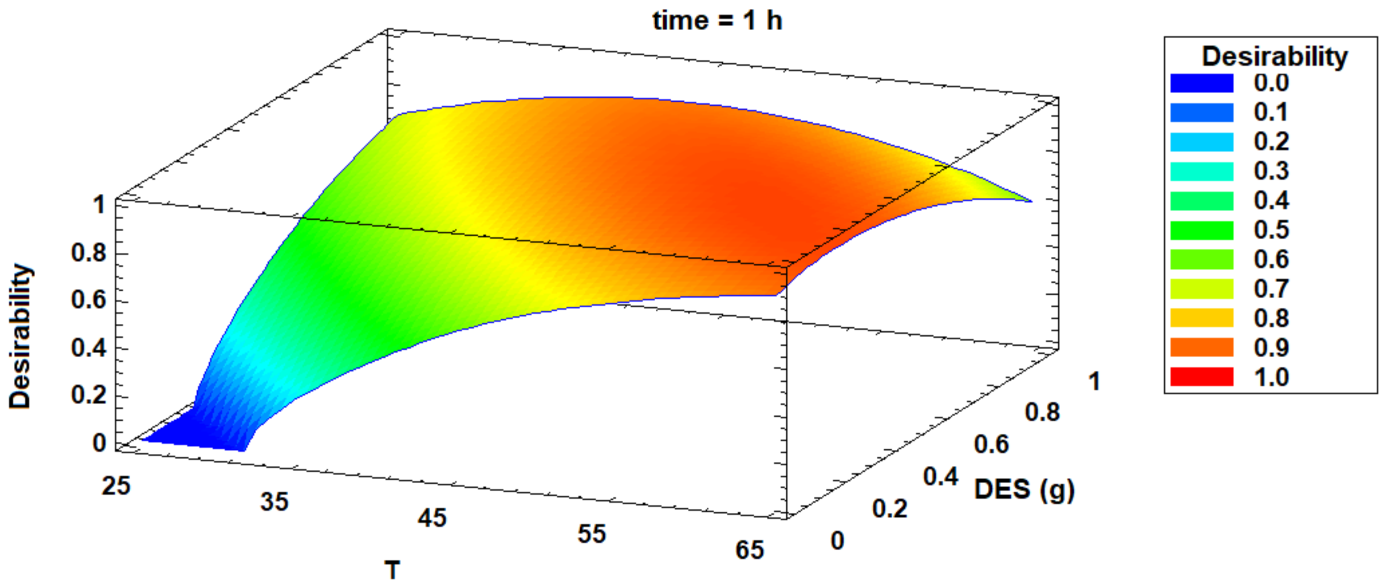

Figure S41. Surface Responding Analysis for 1h of reaction.

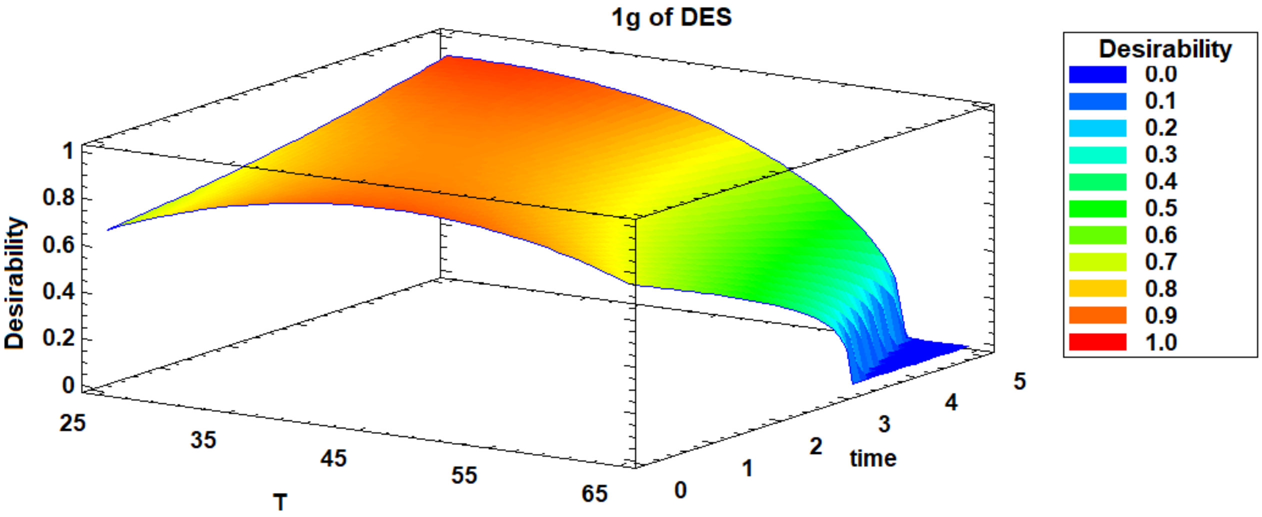

Figure S42. Surface Responding Analysis for 1 g of DES.

References

[1] S. Nejrotti, M. Iannicelli, S. S. Jamil, D. Arnodo, M. Blangetti, C. Prandi, *Green Chem.* **2020**, *22*, 110-117.

[2] Y. Kwon, R. McDonald, F. G. West, *Angew. Chem. Int. Ed.* **2013**, *52*, 8616-8619.
